# Function theory on the annulus in the dp-norm

**DOI:** 10.1007/s00020-025-02814-w

**Published:** 2025-11-03

**Authors:** Jim Agler, Zinaida A. Lykova, N. J. Young

**Affiliations:** 1 Department of Mathematics, University of California at San Diego, San Diego, 92103 USA; 2 School of Mathematics, Statistics and Physics, Newcastle University, Newcastle upon Tyne, NE1 7RU U.K.

**Keywords:** Holomorphic functions, Hilbert space model, annulus, Crouzeix conjecture, Douglas-Paulsen family, 47B99, 30E05, 32A26

## Abstract

In this paper we shall use realization theory, a favourite technique of Rien Kaashoek, to prove new results about a class of holomorphic functions on an annulus

$$ R_\delta {\mathop {=}\limits ^\textrm{def}}\{z\in \mathbb {C}: \delta<|z|<1\}, $$

where $0<\delta <1$. The class of functions in question arises in the early work of R. G. Douglas and V. I. Paulsen on the rational dilation of a Hilbert space operator *T* to a normal operator with spectrum in $\partial R_\delta$. Their work suggested the following norm $\Vert \cdot \Vert _{\textrm{dp}}$ on the space $\textrm{Hol}(R_\delta )$ of holomorphic functions on $R_\delta$,

$$ \Vert \varphi \Vert _{\textrm{dp}} {\mathop {=}\limits ^\textrm{def}} \sup \{ \Vert \varphi (T)\Vert : \Vert T\Vert \le 1, \Vert T^{-1}\Vert \le 1/\delta \ \text {and} \ \sigma (T)\subseteq R_\delta \}. $$

By analogy with the classical Schur class of holomorphic functions $\mathcal {S}$ with supremum norm at most 1 on the disc $\mathbb {D}$, it is natural to consider the *dp-Schur class* $\mathcal {S}_\textrm{dp}$ of holomorphic functions of dp-norm at most 1 on $R_\delta$. Our central result is a Pick interpolation theorem for functions in $\mathcal {S}_\textrm{dp}$ that is analogous to Abrahamse’s Interpolation Theorem for bounded holomorphic functions on a multiply-connected domain. For a tuple $\lambda =(\lambda _1,\dots ,\lambda _n)$ of distinct interpolation nodes in $R_\delta$, we introduce a special set $\mathcal {G}_\textrm{dp}(\lambda )$ of positive definite $n\times n$ matrices, which we call *DP Szegő kernels*. The DP Pick problem $\lambda _j \mapsto z_j, j=1,\dots ,n$, is shown to be solvable if and only if,

$$ {[}(1-{\overline{z}}_i z_j)g_{ij}] \ge 0 \; \text { for all}\; g \in \mathcal {G}_{\textrm{dp}} (\lambda ). $$

We prove further that a solvable DP Pick problem has a solution which is a rational function with a finite-dimensional model, an intriguing result which opens up the possibility of a theory of extremal functions from $\mathcal {S}_\textrm{dp}$ analogous to the theory of finite Blaschke products.

## Introduction

It is our honour to contribute to this memorial issue for Marinus Kaashoek, who was a prolific and influential operator theorist throughout a long career. A constant thread in his research over several decades was the power of realization theory applied to a wide variety of problems in analysis. Among his many contributions in this area we mention his monograph [8], written with his longstanding collaborators Israel Gohberg and Harm Bart, which was an early and influential work in the area, and his more recent papers and book, including [15–17]. Realization theory uses explicit formulae for functions in terms of operators on Hilbert space to prove function-theoretic results. In this paper we continue along the Bart-Gohberg-Kaashoek path by exploiting realization theory to prove new results about a class of holomorphic functions which was first encountered by R. G. Douglas and V. I. Paulsen in a study of rational dilation on the annulus.

For any open set $\Omega$ in the plane, $\textrm{Hol}(\Omega )$ will denote the set of holomorphic functions on $\Omega$ and $\textrm{H}^\infty (\Omega )$ will denote the Banach algebra of bounded holomorphic functions on $\Omega$, equipped with the supremum norm $\Vert \varphi \Vert _{\textrm{H}^\infty (\Omega )}= \sup _{z\in \Omega }|\varphi (z)|$. Let $\mathcal {S}(\Omega )$ denote the class $\{ \varphi \in \textrm{H}^\infty (\Omega ): \Vert \varphi \Vert _{\textrm{H}^\infty (\Omega )} \le 1 \}$. The classical Schur class, $\mathcal {S}$, is the set $\mathcal {S}(\mathbb {D})$.

We recall the extensively-studied Pick interpolation theorem [21] for bounded holomorphic functions on the open unit disc $\mathbb {D}$.

### Theorem 1.1

Let $\lambda _1,\dots ,\lambda _n \in \mathbb {D}$ be distinct and let $z_1,\dots ,z_n\in \mathbb {C}$. There exists $\varphi \in \mathcal {S}$ such that

$$\begin{aligned} \varphi (\lambda _j) = z_j \qquad \text {for} \ j=1,\ldots ,n, \end{aligned}$$

if and only if,

$$ \begin{bmatrix} \displaystyle \frac{1-\overline{z_i} z_j}{1-\overline{\lambda _i}\lambda _j} \end{bmatrix}_{i,j=1}^n \ge 0. $$

Pick interpolation problems, with the unit disc replaced by other domains $\Omega$ in the plane, have also been much studied. In the event that $\Omega$ is a simply connected proper open subset of $\mathbb {C}$, with the aid of the conformal map $F:\Omega \rightarrow \mathbb {D}$, we can convert this problem into a classical Pick problem on $\mathbb {D}$ with interpolation data $F(\lambda _j) \mapsto w_j$ for $j=1,\ldots ,n,$ and then Pick’s theorem gives a criterion for the existence of $\varphi$ in terms of the positivity of the appropriate “Pick matrix”, which here is

$$ \begin{bmatrix} \displaystyle \frac{1-\overline{w_i} w_j}{1-\overline{F(\lambda _i)}F(\lambda _j)} \end{bmatrix}_{i,j=1}^n \ge 0. $$

More generally, the Pick problem on a multiply connected domain was studied in the 1940s by Garabedian [18] and Heins [20]. Later, Sarason [22] and Abrahamse [1] treated the problem in terms of reproducing kernels, an approach that we follow in this paper. Abrahamse’s Theorem gives a solution to the Pick interpolation problem on any bounded domain $\Omega$ in the plane whose boundary consists of finitely many disjoint analytic Jordan curves. He showed that a Pick problem on $\Omega$ can be solved if and only if an infinite collection of Pick matrices are positive semi-definite. In the case of the annulus $R_\delta =\{z\in \mathbb {C}:\delta< |z| < 1\}$, for a tuple $\lambda =(\lambda _1,\dots ,\lambda _n)$ of distinct interpolation nodes in $R_\delta$, Abrahamse [1] described a family $\mathcal {G}(\lambda )$ of positive definite $n \times n$ matrices for which the following statement is true:

### Theorem 1.2

Let $\lambda _1,\dots ,\lambda _n \in R_\delta$ be distinct and let $z_1,\dots ,z_n\in \mathbb {C}$. There exists $\varphi \in \mathcal {S}(R_\delta )$ such that

$$\begin{aligned} \varphi (\lambda _j) = z_j \qquad \text {for} \ j=1,\ldots ,n, \end{aligned}$$

if and only if, for each $g\in \mathcal {G}(\lambda )$,

$$ \begin{bmatrix} (1-\overline{z_i}z_j) g_{ij} \end{bmatrix}_{i,j=1}^n \ge 0. $$

An alternative explicit choice of $\mathcal {G}(\lambda )$ for which Theorem 1.2 is true is described in [2, 22] as follows

$$\mathcal {G}(\lambda )= \{ \begin{bmatrix} g_\rho (\lambda _i,\lambda _j)\end{bmatrix}_{i,j=1}^n: \rho > 0\},$$

where

$$ g_\rho (\lambda _i,\lambda _j) = \sum _{m=-\infty }^\infty \frac{({\overline{\lambda }}_i \lambda _j)^m}{\rho + \delta ^{2m}},\quad \text {for}\ 1\le i,j \le n. $$

Another natural variant of Pick’s problem arises if one replaces the supremum norm on $\textrm{Hol}(\Omega )$ by a different norm. For example, consider the *Dirichlet space* $\mathcal {D}$ of holomorphic functions *f* on $\mathbb {D}$ such that $f'$ is square integrable with respect to area measure on $\mathbb {D}$, with pointwise operations and the norm

$$ \Vert f\Vert _\mathcal {D}^2 = \sum _{n=0}^\infty (n+1) |{\hat{f}}(n)|^2 = \Vert f\Vert _{H^2}^2 + \int _\mathbb {D}|f'(z)|^2 dm(z), $$

where *m* denotes area measure on the disc. The Dirichlet space is a Hilbert function space on $\mathbb {D}$ with reproducing kernel

$$ k_{\mathcal {D}}(\lambda ,\mu )= -\frac{1}{{\overline{\mu }} \lambda }\log (1-{\overline{\mu }}\lambda ). $$

The Pick-type interpolation problem appropriate to this Hilbert function space is expressed in terms of its *multiplier space* $\mathcal {M}(\mathcal {D})$, which is defined to be the space of functions $\varphi$ on $\mathbb {D}$ such that $\varphi f \in \mathcal {D}$ for every $f\in \mathcal {D}$, with pointwise operations and the *multiplier norm*

$$ \Vert \varphi \Vert _{\mathcal {M}(\mathcal {D})} = \sup \{\Vert \varphi f\Vert _\mathcal {D}: f\in \mathcal {D}, \Vert f\Vert _{\mathcal {D}} \le 1\}. $$

In this setting the corresponding Pick interpolation theorem is the following [3, Corollary 7.41]:

### Theorem 1.3

Let $\lambda _1,\dots ,\lambda _n \in \mathbb {D}$ be distinct and let $z_1,\dots ,z_n\in \mathbb {C}$. There exists $\varphi \in \mathcal {M}(\mathcal {D})$ such that $\Vert \varphi \Vert _{\mathcal {M}(\mathcal {D})} \le 1$ and

$$\begin{aligned} \varphi (\lambda _j) = z_j \qquad \text {for} \ j=1,\ldots ,n, \end{aligned}$$

if and only if

$$ \begin{bmatrix} (1- z_i \overline{z_j}) k_\mathcal {D}(\lambda _i,\lambda _j) \end{bmatrix}_{i,j=1}^n \ge 0. $$

An account of Pick theorems in the context of sundry different Hilbert function spaces, including $\mathcal {D},$ may be found in the book [3].

In this paper we will deviate from the supremum norm on $\textrm{Hol}(R_\delta ), \delta \in (0,1)$. An operator *X* on a Hilbert space is called a *Douglas-Paulsen operator with parameter* $\delta$ if $\Vert X\Vert \le 1$ and $\Vert X^{-1}\Vert \le 1/\delta$, see [14]. The *Douglas-Paulsen family*, $\mathcal {F}_{\textrm{dp}}(\delta )$, is the class of Douglas-Paulsen operators *X* with parameter $\delta$ such that $\sigma (X)\subseteq R_\delta$. We consider the *Douglas-Paulsen norm*^1^

1.4 $$\begin{aligned} \Vert \varphi \Vert _{\textrm{dp}} = \sup _{X\in \mathcal {F}_{\textrm{dp}}(\delta ) }\Vert \varphi (X) \Vert , \end{aligned}$$

defined for $\varphi \in \textrm{Hol}(R_\delta )$. There is no guarantee that the quantity defined by equation (1.4) is finite. Accordingly, we introduce the associated Banach algebra

$$ \textrm{H}^\infty _{\textrm{dp}}(R_\delta )=\{ \varphi \in \textrm{Hol}(R_\delta ) \, : \, \Vert \varphi \Vert _{\textrm{dp}}<\infty \}. $$

In addition, we introduce the *dp-Schur class*, $\mathcal {S}_{\textrm{dp}}$^2^, which is the set of functions $\varphi \in \textrm{Hol}(R_\delta )$ such that $\Vert \varphi \Vert _{\textrm{dp} }\le 1.$

An important step in the Douglas-Paulsen theory was the following estimate. If *X* is a Douglas-Paulsen operator with parameter $\delta$, $\sigma (X)\subseteq R_{\delta }$ and $\varphi$ is a bounded holomorphic matrix-valued function on $R_{\delta }$ then

1.5 $$\begin{aligned} \Vert \varphi (X)\Vert \le \left( 2+\frac{1+\delta }{1-\delta } \right) \sup _{z\in R_\delta } \Vert \varphi (z)\Vert . \end{aligned}$$

Hence, we see from equations (1.4) and (1.5) that

$$\Vert \varphi \Vert _\textrm{dp} \le \left( 2+\frac{1+\delta }{1-\delta } \right) \Vert \varphi \Vert _{\textrm{H}^\infty (R_\delta )}$$

for $\varphi \in \textrm{Hol}(R_\delta )$. On the other hand, see Remark 2.7, $\Vert \varphi \Vert _{\textrm{H}^\infty (R_\delta )} \le \Vert \varphi \Vert _\textrm{dp}$, and so the dp and supremum norms on $\textrm{Hol}(R_\delta )$ are equivalent. Thus,

$$ \textrm{H}^\infty (R_\delta ) = \textrm{H}_\textrm{dp}^\infty (R_\delta ) $$

as sets. However, the reader should be aware that

$$\Vert \cdot \Vert _\textrm{dp} \ne \Vert \cdot \Vert _{\textrm{H}^\infty (R_\delta )} \ \text {and therefore} \ \mathcal {S}_{\textrm{dp}}\ne \mathcal {S}(R_\delta ), $$

a fact that Example 2.9 below demonstrates.

The power of inequality (1.5) is that it holds for all *matrix-valued* functions $\varphi$, a fact which allowed Douglas and Paulsen to show that if $T \in \mathcal {B}(\mathcal {H})$ is a Douglas-Paulsen operator, then there exists an invertible $S \in \mathcal {B}(\mathcal {H})$ such that

1.6 $$\begin{aligned} \Vert S\Vert \Vert S^{-1}\Vert \le 2+\frac{1+\delta }{1-\delta } \end{aligned}$$

and $S T S^{-1}$ dilates to a normal operator with spectrum contained in the boundary $\partial R_\delta$. This result is a kind of Nagy dilation theorem for the annulus. In the *scalar case* a slightly stronger result than the inequality (1.5) had been obtained earlier by A. Shields [24, Proposition 23], with the smaller constant $2+\sqrt{\frac{1+\delta }{1-\delta }}$ on the right hand side. Shields asked whether the constant $2+\sqrt{\frac{1+\delta }{1-\delta }}$ could be replaced by a quantity that remains bounded as $\delta \rightarrow 1$. This question was answered in the affirmative by C. Badea, B. Beckermann and M. Crouzeix [7] and subsequently the better constant $1+\sqrt{2}$ was established by M. Crouzeix and A. Greenbaum [11].

Corresponding to the dp-Schur class there is a natural variant of the classical Pick interpolation problem, which we call the *DP Pick problem*: given *n* distinct points $\lambda _1,\ldots ,\lambda _n$ in $R_\delta$ and $z_1,\ldots ,z_n \in \mathbb {C}$, does there exist a function $\varphi \in \textrm{H}^\infty _{\textrm{dp}}(R_\delta )$ with $\Vert \varphi \Vert _{\textrm{dp}} \le 1$ such that

1.7 $$\begin{aligned} \varphi (\lambda _j) = z_j \qquad \text {for} \ j=1,\ldots ,n ? \end{aligned}$$

We shall show that there is a solvability criterion for this problem which is parallel to Abrahamse’s Theorem, but with $\mathcal {G}(\lambda )$ replaced by a collection $\mathcal {G}_\textrm{dp}(\lambda )$ of kernels, which we now define.

### Definition 1.8

Let $\lambda _1, \dots , \lambda _n$ be *n* distinct points in $R_\delta$ and let $\lambda =(\lambda _1,\dots ,\lambda _n)$. A *DP Szegő kernel* for the *n*-tuple $\lambda$ is a positive definite $n \times n$ matrix $g = [g_{ij}]$ such that

1.9 $$\begin{aligned} {[}(1-{\overline{\lambda }}_i \lambda _j)g_{ij}] \ge 0\ \ \text{ and } \ \ \left[ \left( 1-\frac{\delta }{\overline{\lambda _i}} \frac{\delta }{\lambda _j}\right) g_{ij}\right] \ge 0. \end{aligned}$$

The set of all DP Szegő kernels for the *n*-tuple $\lambda$ will be denoted by $\mathcal {G}_\textrm{dp}(\lambda )$.

We observe that $\mathcal {G}_\textrm{dp}(\lambda )$ consists of the gramians $[\langle e_j,e_i\rangle ]_{i,j=1}^n$ for all bases $e_1,\dots ,e_n$ of an *n*-dimensional Hilbert space $\mathcal {H}$ such that the operator *T* on $\mathcal {H}$ defined by $Te_j= \lambda _je_j$ for $j=1,\dots ,n$ is a Douglas-Paulsen operator. This and related facts are described in Section 4.

The Pick interpolation theorem for the dp-norm on $\textrm{Hol}(R_\delta )$ is the following statement (which is Theorem 5.2 from the body of the paper).

### Theorem 1.10

Let $\lambda _1,\dots ,\lambda _n \in R_\delta$ be distinct and let $z_1,\dots ,z_n\in \mathbb {C}$. There exists $\varphi \in \mathcal {S}_{\textrm{dp}}$ such that

$$\begin{aligned} \varphi (\lambda _j) = z_j \qquad \text {for} \ j=1,\ldots ,n, \end{aligned}$$

if and only if, for all $g \in \mathcal {G}_{\textrm{dp}} (\lambda )$,

1.11 $$\begin{aligned} {[}(1-{\overline{z}}_i z_j)g_{ij}] \ge 0. \end{aligned}$$

In Section 2 we compare the dp norm and the sup norm of a function in $\textrm{Hol}(R_\delta )$ and we point out a connection to the Crouzeix conjecture. In Section 3 we review the theory of models and realizations of holomorphic functions on $R_\delta$ with dp-norm at most 1, see Theorem 3.8. In Section 4 we introduce DP-Szegő kernels on an *n*-tuple of points in $R_\delta$ and elaborate their relation to the Douglas-Paulsen class. In Section 5 we recall another approach to the solution of DP Pick problems given in [6, Theorem 9.46], and we show that solvable DP Pick problems have *rational* solutions. In Section 6 we consider an extremally solvable DP Pick problem $\lambda _j \mapsto z_j$ for $j=1,\dots ,n$, and show that, for such a problem there is a rational solution $\varphi \in \mathcal {S}_{\textrm{dp}}$ and there exists a Douglas-Paulsen operator *T* with parameter $\delta$ which acts on an *n*-dimensional Hilbert space with $\sigma (T)=\{\lambda _1,\dots ,\lambda _n\}$ such that $\Vert \varphi \Vert _{\textrm{dp}} = \Vert \varphi (T) \Vert =1,$ see Theorem 6.13.

## The dp and sup norms on $\textrm{Hol}(\mathbb {D})$ and $\textrm{Hol}(R_\delta )$

In this section we describe connections between the Banach algebra $\textrm{H}^\infty _{\textrm{dp}}(R_\delta )$ and the Crouzeix conjecture. We will prove in Proposition 2.11 that there is a large class of functions $\varphi \in \textrm{Hol}(R_\delta )$, such that

$$ \Vert \varphi \Vert _{\textrm{dp}} = \Vert \varphi \Vert _{\textrm{H}^\infty (R_\delta )}. $$

In Example 2.9 below we show that the last relation fails to hold for the function $\varphi \in \textrm{Hol}(R_\delta )$ defined by $\varphi (z)=z +\frac{\delta }{z}$ for $z\in R_\delta$. In fact $\varphi$ satisfies

$$ \Vert \varphi \Vert _{\textrm{dp}} = 2 \ \text {and}\ \Vert \varphi \Vert _{\textrm{H}^\infty (R_\delta )} = 1+\delta . $$

By an *elliptical domain* we shall mean the domain in the complex plane bounded by an ellipse. As a standard elliptical domain we take the set

2.1 $$\begin{aligned} G_\delta {\mathop {=}\limits ^\textrm{def}}\{x+iy:x,y\in \mathbb {R}, \frac{x^2}{(1+\delta )^2} + \frac{y^2}{(1-\delta )^2} < 1\}, \end{aligned}$$

for some $\delta$ such that $0\le \delta <1$. Note that any elliptical domain can be identified via an affine self-map of the plane with an elliptical domain of the form $G_\delta$ for some $\delta \in [0,1)$.

In this paper all Hilbert spaces are complex Hilbert spaces. For a complex Hilbert space $\mathcal {H}$ we denote by $\mathcal {B}(\mathcal {H})$ the space of bounded operators on $\mathcal {H}$. If $T\in \mathcal {B}(\mathcal {H})$, then *W*(*T*), *the numerical range of T*, is defined by the formula

$$ W(T) = \{ \langle Tx,x\rangle _\mathcal {H} \, : \, x\in \mathcal {H}, \Vert x \Vert =1 \}. $$

The *B. and F. Delyon family*, $\mathcal {F}_{\textrm{bfd}}(C)$, corresponding to an open bounded convex set *C* in $\mathbb {C}$ is the class of operators *T* such that the closure of the numerical range of *T*, $\overline{W(T)}$, is contained in *C*. By [19, Theorem 1.2-1], the spectrum $\sigma (T)$ of an operator *T* is contained in $\overline{W(T)}$, and so, by the Riesz-Dunford functional calculus, $\varphi (T)$ is defined for all $\varphi \in \textrm{Hol}(C)$ and $T\in \mathcal {F}_{\textrm{bfd}}(C)$. Therefore, we may consider the calcular norm^3^

2.2 $$\begin{aligned} \Vert \varphi \Vert _{\mathcal {F}_{\textrm{bfd}}(C)}=\sup _{T\in \mathcal {F}_{\textrm{bfd}}(C)}\Vert \varphi (T) \Vert , \end{aligned}$$

defined for $\varphi \in \textrm{Hol}(C)$, and the associated Banach algebra

$$ \textrm{H}^\infty _{\textrm{bfd}}(C)=\{ \varphi \in \textrm{Hol}(C) \, : \, \Vert \varphi \Vert _{\mathcal {F}_{\textrm{bfd}}(C)} <\infty \}. $$

In this paper the convex set *C* will always be $G_\delta$, and so we abbreviate the notation to $\Vert \cdot \Vert _{\textrm{bfd}}$ in place of $\Vert \cdot \Vert _{\mathcal {F}_{\textrm{bfd}}(G_\delta )}$. Thus

2.3 $$\begin{aligned} \Vert \varphi \Vert _{\textrm{bfd}}=\sup _{T\in \mathcal {F}_{\textrm{bfd}}(G_\delta )}\Vert \varphi (T) \Vert , \end{aligned}$$

defined for $\varphi \in \textrm{Hol}(G_\delta )$. In addition we introduce the *bfd-Schur class*, $\mathcal {S}_{\textrm{bfd}}$, of functions on $G_\delta$, which is the set of functions $f \in \textrm{Hol}(G_\delta )$ such that $\Vert f\Vert _{\textrm{bfd} }\le 1.$^4^ The bfd-norm is named in recognition of a celebrated theorem [13] of the brothers B. and F. Delyon, which states that, if *p* is a polynomial, $\mathcal {H}$ is a Hilbert space and $T\in \mathcal {B}(\mathcal {H})$ then

$$ \Vert p(T)\Vert \le \kappa (W(T))\Vert p\Vert _{W(T)}, $$

where $\Vert \cdot \Vert _{W(T)}$ denotes the supremum norm on *W*(*T*), and, for any bounded convex set *C* in $\mathbb {C}$, $\kappa (C)$ is defined by

$$ \kappa (C) = 3+\left( \frac{2\pi (\textrm{diam}(C))^2}{\textrm{area}(C)}\right) ^3. $$

Let us write

$$ K(\mathcal {F}_{\textrm{bfd}}(C)) = \sup _{\varphi \in \textrm{Hol}(C): \Vert \varphi \Vert _{\textrm{H}^\infty (C)} \le 1} \Vert \varphi \Vert _{\mathcal {F}_{\textrm{bfd}}(C)}, $$

and the Crouzeix universal constant

$$ K_{\textrm{bfd}} =\sup \{ K(\mathcal {F}_{\textrm{bfd}}(C)): \ C \ \text {is a bounded convex set in } \ \mathbb {C}\}. $$

In [9], Crouzeix proved $K_{\textrm{bfd}} \le 12$ and conjectured that $K_{\textrm{bfd}} = 2$. Subsequently Crouzeix and Palencia [10] proved that $K_{\textrm{bfd}} \le 1+\sqrt{2}$. Still more recently Crouzeix and Kressner [12] showed that *W*(*T*) is a *complete* $(1+\sqrt{2})$-spectral set for *T*.

Let $\pi : R_\delta \rightarrow G_\delta$ be defined by $\pi (z)=z +\frac{\delta }{z}$, $z \in R_\delta$. Now observe that if $\varphi \in \textrm{Hol}(G_\delta )$ then we may define $\pi ^\sharp (\varphi ) \in \textrm{Hol}(R_\delta )$ by the formula

$$ \pi ^\sharp (\varphi )(\lambda )=\varphi (\pi (\lambda )) \qquad \text { for all } \lambda \in R_\delta . $$

We record the following simple fact from complex analysis without proof.

### Lemma 2.4

Let $\delta \in (0,1)$ and let $\psi \in \textrm{Hol}(R_\delta )$. Then $\psi \in {{\,\textrm{ran}\,}}\pi ^\sharp$ if and only if $\psi$ is *symmetric* with respect to the involution $\lambda \mapsto \delta /\lambda$ of $R_\delta$, that is, if and only if $\psi$ satisfies

$$ \psi (\delta /\lambda )=\psi (\lambda ) $$

for all $$\lambda \in R_\delta $$.

The following result, which is [4, Theorem 11.25], gives an intimate connection between the $\Vert \cdot \Vert _{\textrm{dp}}$ and $\Vert \cdot \Vert _{\textrm{bfd}}$ norms.

### Theorem 2.5

Let $\delta \in (0,1)$. The mapping $\pi ^\sharp$ is an isometric isomorphism from $\textrm{H}^\infty _{\textrm{bfd}}(G_\delta )$ onto the set of symmetric functions with respect to the involution $\lambda \mapsto \delta /\lambda$ in $\textrm{H}^\infty _{\textrm{dp}}(R_\delta )$, so that, for all $\varphi \in \textrm{Hol}(G_\delta )$,

2.6 $$\begin{aligned} \Vert \varphi \Vert _{\textrm{bfd}} = \Vert \varphi \circ \pi \Vert _{\textrm{dp}}. \end{aligned}$$

### Remark 2.7

One can see that, for $\varphi \in \textrm{Hol}(R_\delta )$,

2.8 $$\begin{aligned} \Vert \varphi \Vert _{\textrm{dp}}&= \sup _{X\in \mathcal {F}_{\textrm{dp}}(\delta ) }\Vert \varphi (X) \Vert \nonumber \\&\ge \sup _{X\in \mathcal {F}_{\textrm{dp}}(\delta ) \ \text {and} \ X \ \text { is a scalar operator} \ }\Vert \varphi (X) \Vert \nonumber \\&= \sup _{\lambda \in R_\delta }{|\varphi (\lambda )|} =\Vert \varphi \Vert _{\textrm{H}^\infty (R_\delta )}. \end{aligned}$$

### Example 2.9

Consider the function $f\in \textrm{Hol}(R_\delta )$ defined by $f(z)=z +\frac{\delta }{z}$. Then

$$ \Vert f \Vert _{\textrm{dp}} = 2 \ \text {and}\ \Vert f \Vert _{\textrm{H}^\infty (R_\delta )} = 1+\delta . $$

Moreover, the Crouzeix universal constant $K_{\textrm{bfd}} \ge 2$.

### Proof

If $\varphi (z) = z$ for $z \in G_\delta$ and $\pi : R_\delta \rightarrow G_\delta$ is defined by $\pi (z)=z +\frac{\delta }{z}$, then

$$\begin{aligned} \varphi \circ \pi (z)=z +\frac{\delta }{z} =f(z) \ \text { for} \ z \in R_\delta . \end{aligned}$$

By Theorem 2.5,

$$\begin{aligned} \Vert \varphi \Vert _{\textrm{bfd}} = \Vert \varphi \circ \pi \Vert _{\textrm{dp}}. \end{aligned}$$

By [5, Example 4.26], $\Vert \varphi \Vert _{\textrm{bfd}} = 2$. Therefore,

$$ \Vert f \Vert _{\textrm{dp}} = \Vert \varphi \circ \pi \Vert _{\textrm{dp}}= \Vert \varphi \Vert _{\textrm{bfd}} = 2. $$

Note that

$$ \Vert f \Vert _{\textrm{H}^\infty (R_\delta )} =\sup _{z \in R_\delta } \left| z +\frac{\delta }{z} \right| = 1+\delta . $$

Note that $\varphi (z)=z$ has bfd-norm equal to 2 and sup norm on $G_\delta$ equal to $1+\delta$. Hence the Crouzeix universal constant $K_{\textrm{bfd}} \ge 2$. $\square$

### Remark 2.10

In [23] G. Tsikalas proved a result about the annulus as a *K*-spectral set. We restate his result in the notation of this paper as follows. Let $K(\delta )$ denote the smallest constant such that $R_\delta$ is a $K(\delta )$-spectral set for any bounded linear operator $T \in \mathcal {F}_{\textrm{dp}}(\delta )$. He used the functions $g_n$ in $\textrm{Hol}(R_\delta )$ defined by

$$g_n(z) = \frac{\delta ^n}{z^n} + z^n, \quad \text {for}\ n=1, 2, \dots ,$$

to show that $K(\delta ) \ge 2$, for all $\delta \in (0,1)$.

### Proposition 2.11

If $$\varphi \in \textrm{Hol}(\mathbb {D})$$, then

2.12 $$\begin{aligned} \Vert \varphi \Vert _{\textrm{dp}}= &   \sup _{X\in \mathcal {F}_{\textrm{dp}}(\delta ) }\Vert \varphi (X) \Vert \nonumber \\= &   \Vert \varphi \Vert _{\textrm{H}^\infty (R_\delta )} =\Vert \varphi \Vert _{\textrm{H}^\infty (\mathbb {D})}. \end{aligned}$$

### Proof

By the definition of the dp-norm,

2.13 $$\begin{aligned} \Vert \varphi \Vert _{\textrm{dp}}= &   \sup _{X\in \mathcal {F}_{\textrm{dp}}(\delta )}\Vert \varphi (X) \Vert \nonumber \\\le &   \sup _{\Vert X\Vert \le 1} \Vert \varphi (X) \Vert \quad \text {by the definition of }\mathcal {F}_{\textrm{dp}}(\delta ) \nonumber \\= &   \Vert \varphi \Vert _{\textrm{H}^\infty (\mathbb {D})}. \quad \text {by von Neumann's inequality} \end{aligned}$$

By the Maximum principle, for $\varphi \in \textrm{Hol}(\mathbb {D})$,

2.14 $$\begin{aligned} \Vert \varphi \Vert _{\textrm{H}^\infty (R_\delta )}= \Vert \varphi \Vert _{\textrm{H}^\infty (\mathbb {D})}. \end{aligned}$$

By inequality (2.8), $\Vert \varphi \Vert _{\textrm{dp}} \ge \Vert \varphi \Vert _{\textrm{H}^\infty (R_\delta )}$ and, by inequality (2.13), $\Vert \varphi \Vert _{\textrm{dp}} \le \Vert \varphi \Vert _{\textrm{H}^\infty (\mathbb {D})}$, and so

2.15 $$\begin{aligned} \Vert \varphi \Vert _{\textrm{dp}} =\Vert \varphi \Vert _{\textrm{H}^\infty (\mathbb {D})}. \end{aligned}$$

Therefore, the equalities (2.12) hold. $\square$

## Models and realizations of holomorphic functions on $R_\delta$

In this section we review some known results on the function theory of holomorphic functions in the dp-norm on an annulus. The models and realizations of holomorphic functions $\varphi :R_\delta \rightarrow \mathbb {C}$ such that $\Vert \varphi \Vert _{\textrm{dp}} \le 1$ are presented in [6, Theorem 9.46]. The theorem states the following.

### Theorem 3.1

Let $\delta \in (0,1)$. Let $\varphi :R_\delta \rightarrow \mathbb {C}$ be holomorphic and satisfy $\Vert \varphi \Vert _{\textrm{dp}} \le 1$. There exists a $\textrm{dp}$-model $(\mathcal {N},v)$ of $\varphi$ with parameter $\delta$, in the sense that there are Hilbert spaces $\mathcal {N}^+,\mathcal {N}^-$ and an ordered pair $v=(v^+,v^-)$ of holomorphic functions, where $v^+:R_\delta \rightarrow \mathcal {N}^+$ and $v^-:R_\delta \rightarrow \mathcal {N}^-$ satisfy, for all $z,w \in R_\delta$,

$$ 1-\overline{\varphi (w)}\varphi (z) = (1-{\overline{w}} z)\langle v^+(z),v^+(w)\rangle _{\mathcal {N}^+} + ({\overline{w}} z-\delta ^2)\langle v^-(z),v^-(w)\rangle _{\mathcal {N}^-}. $$

### Definition 3.2

A *positive semi-definite function* on a set *X* is a function $A: X\times X \rightarrow \mathbb {C}$ such that, for any positive integer *n* and any points $x_1,\dots ,x_n \in X$, the $n\times n$ matrix $[A(x_j,x_i)]_{i,j=1}^n$ is positive semi-definite.

We shall write

$$ {[}A(x,y)] \ge 0, \ \text {for all} \ x,y\in X, $$

to mean that *A* is a positive semi-definite function on *X*.

### Theorem 3.3

$\varphi \in \mathcal {S}_{\textrm{dp}}$ if and only if there exist a pair of positive semi-definite functions *A* and *B* on $R_\delta$ such that

3.4 $$\begin{aligned} 1-\overline{\varphi (\mu )}\varphi (\lambda )= (1-{\overline{\mu }} \lambda )A(\lambda ,\mu )+ (1-\frac{\delta }{\overline{\mu }}\frac{\delta }{\lambda })B(\lambda ,\mu ) \end{aligned}$$

for all $$\lambda ,\mu \in R_\delta $$.

### Proof

For a proof see Definition 9.44 and Theorem 9.46 in [6]. $\square$

Recall Moore’s Theorem [6, Theorem 2.5]: if $\Omega$ is a set and $A:\Omega \times \Omega \rightarrow \mathbb {C}$ is a function, then *A* is a positive semi-definite function on $\Omega$ if and only if there exists a Hilbert space $\mathcal {M}$ and a function $u:\Omega \rightarrow \mathcal {M}$ satisfying

3.5 $$\begin{aligned} A(\lambda ,\mu )=\langle u(\lambda ),u(\mu )\rangle _\mathcal {M}\end{aligned}$$

for all $\lambda ,\mu \in \Omega$. Thus, if *A* and *B* are as in equation (3.4), we may choose Hilbert spaces $\mathcal {M}_1$ and $\mathcal {M}_2$ such that

$$ A(\lambda ,\mu )=\langle u_1(\lambda ),u_1(\mu )\rangle _{\mathcal {M}_1}\ \text { and }\ B(\lambda ,\mu )=\langle u_2(\lambda ),u_2(\mu )\rangle _{\mathcal {M}_2} $$

for all $\lambda ,\mu \in R_\delta$. If we then let $\mathcal {M}=\mathcal {M}_1 \oplus \mathcal {M}_2$ and define $E:R_\delta \rightarrow \mathcal {B}(\mathcal {M})$ and $u:R_\delta \rightarrow \mathcal {M}$ by the formulae

3.6 $$\begin{aligned} E(\lambda )=\begin{bmatrix}\lambda &  0\\ 0 &  \frac{\delta }{\lambda }\end{bmatrix}\ \text { and }\ u(\lambda )=\begin{bmatrix}u_1(\lambda )\\ u_2(\lambda )\end{bmatrix},\qquad \text {for}\ \lambda \in R_\delta , \end{aligned}$$

then the relation (3.4) becomes the formula

3.7 $$\begin{aligned} 1-\overline{\varphi (\mu )}\varphi (\lambda ) = \Big \langle \, \big (1-E(\mu )^*E(\lambda )\big )\ u(\lambda )\, ,u(\mu )\, \Big \rangle _\mathcal {M}\ \text {for}\ \lambda ,\mu \in R_\delta . \end{aligned}$$

When *A* is positive semi-definite, let us agree to say that *A* *has finite rank* if $\mathcal {M}$ in the formula (3.5) can be chosen to have finite dimension. In this case, we may define ${{\,\textrm{rank}\,}}(A)$ by setting

$$ {{\,\textrm{rank}\,}}(A) = \dim \mathcal {M}$$

where $\mathcal {M}$ satisfying (3.5) is chosen to have minimal dimension.^5^

The following theorem is stated as [6, Theorem 9.54]. For the convenience of the reader we shall give a full proof here.

### Theorem 3.8

**A realization formula.** Let $\varphi \in \mathcal {S}_{\textrm{dp}}(R_\delta )$. If $(\mathcal {M}, u)$ is a model for $\varphi$ then there exists a unitary operator $L \in \mathcal {B}(\mathbb {C}\oplus \mathcal {M})$ such that if we decompose *L* as a block operator matrix

3.9 $$\begin{aligned} L=\begin{bmatrix}a& 1\otimes \beta \\ \gamma \otimes 1&  D\end{bmatrix}, \end{aligned}$$

where $a \in \mathbb {C}$, $\beta \in \mathcal {M}$, $\gamma \in \mathcal {M}$, and $D \in \mathcal {B}(\mathcal {M})$, then

3.10 $$\begin{aligned} \varphi (\lambda )=a + \Big \langle \, E(\lambda )\big (1-DE(\lambda )\big )^{-1}\ \gamma \, ,\beta \, \Big \rangle _\mathcal {M},\qquad \text {for all}\ \lambda \in R_\delta . \end{aligned}$$

Conversely, if $a \in \mathbb {C}$, $\beta \in \mathcal {M}$, $\gamma \in \mathcal {M}$, and $D \in \mathcal {B}(\mathcal {M})$ are such that *L* as defined by equation (3.9) is unitary and if $\varphi$ is given by equation (3.10) and $u:R_\delta \rightarrow \mathcal {M}$ is defined by

3.11 $$\begin{aligned} u(\lambda )=\big (1-DE(\lambda )\big )^{-1}\gamma ,\qquad \text {for}\ \lambda \in R_\delta , \end{aligned}$$

then $(\mathcal {M},u)$ is a model for $\varphi$.

### Proof

Let $(\mathcal {M},u)$ be a model for $\varphi$. As explained in Theorem 3.1, it means that there exist Hilbert spaces $\mathcal {N}^+$ and $\mathcal {N}^-$ and maps $v^+: R_\delta \rightarrow \mathcal {N}^+, v^-:R_\delta \rightarrow \mathcal {N}^-$ such that, for all $\lambda ,\mu \in R_\delta$,

$$ 1- \overline{\varphi (\mu )}\varphi (\lambda ) = (1-{\overline{\mu }}\lambda )\langle v^+(\lambda ),v^+(\mu )\rangle _{\mathcal {N}^+} + ({\overline{\mu }}\lambda -\delta ^2)\langle v^-(\lambda ),v^-(\mu )\rangle _{\mathcal {N}^-}. $$

Reshuffle this relation to

$$\begin{aligned} 1+&\langle \lambda v^+(\lambda ),\mu v^+(\mu )\rangle _{\mathcal {N}^+} + \langle \delta v^-(\lambda ),\delta v^-(\mu )\rangle _{\mathcal {N}^-} \\&= \overline{\varphi (\mu )}\varphi (\lambda ) + \langle v^+ (\lambda ),v^+ (\mu )\rangle _{\mathcal {N}^+} +\langle \lambda v^- (\lambda ),\mu v^-(\mu )\rangle _{\mathcal {N}^-}, \end{aligned}$$

and notice that this equation amounts to saying that the following families of vectors in $\mathbb {C}\oplus \mathcal {N}$, where $\mathcal {N}{\mathop {=}\limits ^\textrm{def}}\mathcal {N}^+ \oplus \mathcal {N}^-$,

$$ \begin{pmatrix} 1 \\ \lambda v^+(\lambda ) \\ \delta v^-(\lambda ) \end{pmatrix}_{\lambda \in R_\delta } \quad \text {and}\quad \begin{pmatrix} \varphi (\lambda )\\ v^+(\lambda ) \\ \lambda v^-(\lambda ) \end{pmatrix}_{\lambda \in R_\delta } $$

have the same gramian. Let the closed linear spans of these two families be $\mathcal {X}$ and $\mathcal {Y}$ respectively. By the Lurking Isometry Lemma [6, Lemma 2.18] there exists a linear isometry $L:\mathcal {X}\rightarrow \mathcal {Y}$ such that

3.12 $$\begin{aligned} L\begin{pmatrix} 1 \\ \lambda v^+(\lambda ) \\ \delta v^-(\lambda ) \end{pmatrix} = \begin{pmatrix} \varphi (\lambda )\\ v^+(\lambda ) \\ \lambda v^-(\lambda ) \end{pmatrix} \end{aligned}$$

for all $\lambda \in R_\delta$. Since both $\mathcal {X}$ and $\mathcal {Y}$ are subspaces of $\mathbb {C}\oplus \mathcal {N}$, we may extend *L* (possibly after enlarging the space $\mathcal {N}$) to a unitary operator $L:\mathcal {N}\rightarrow \mathcal {N}$ (see the discussion in [6, Remark 2.31] for this step). Write *L* as a block operator matrix

3.13 $$\begin{aligned} L \sim \begin{pmatrix} a &  1\otimes \beta \\ \gamma \otimes 1&  D \end{pmatrix} \end{aligned}$$

with respect to the orthogonal decomposition $\mathbb {C}\oplus (\mathcal {N}^+\oplus \mathcal {N}^-)$ of $\mathbb {C}\oplus \mathcal {N}$ and define a map $u:R_\delta \rightarrow \mathcal {N}$ by

$$ u(\lambda ) = \begin{pmatrix} v^+(\lambda ) \\ \lambda v^-(\lambda ) \end{pmatrix}. $$

Then equation (3.12) yields the relations

3.14 $$\begin{aligned}  &   a + \langle E(\lambda ) u(\lambda ),\beta \rangle _{\mathcal {N}}= \varphi (\lambda ) \end{aligned}$$

3.15 $$\begin{aligned}  &   \gamma + DE(\lambda )u(\lambda ) = u(\lambda ), \end{aligned}$$

where $E(\lambda )$ is given by equation (3.6). Since $\Vert D\Vert \le 1$ and

$$\Vert E(\lambda )\Vert = \max {\left\{ |\lambda |, \frac{\delta }{|\lambda |} \right\} } < 1 \ \text { for all} \ \lambda \in R_\delta ,$$

it follows that $1-DE(\lambda )$ is invertible for $\lambda \in R_\delta$, and hence

$$\begin{aligned} u(\lambda )&= (1-DE(\lambda ))^{-1}\gamma , \\ \varphi (\lambda )&= a + \langle E(\lambda )(1-DE(\lambda ))^{-1}\gamma ,\beta \rangle \end{aligned}$$

for all $\lambda \in R_\delta$, which is the desired realization formula (3.10).

Conversely, suppose that $a,\beta ,\gamma , D$ are such that *L* given by equation (3.13) is a unitary operator on $\mathbb {C}\oplus \mathcal {N}$ and that $\varphi$ is the function on $R_\delta$ defined by equation (3.10). Since $1-DE(\lambda )$ is invertible for all $\lambda \in R_\delta$ we may define a mapping $u:R_\delta \rightarrow \mathcal {N}$ by equation (3.11). Then the equations (3.143.15) hold. They may be written in the form

$$ L\begin{bmatrix} 1\\ E(\lambda ) u(\lambda ) \otimes 1 \end{bmatrix} = \begin{bmatrix} \varphi (\lambda )\otimes 1 \\ u(\lambda )\otimes 1 \end{bmatrix} \ \text {for}\ \lambda \in R_\delta . $$

Thus, for any $\mu \in R_\delta$,

$$ \begin{bmatrix} 1&1\otimes E(\mu )u(\mu ) \end{bmatrix} L^* = \begin{bmatrix} 1\otimes \varphi (\mu )&1\otimes u(\mu ) \end{bmatrix}. $$

Multiply the last two displayed equations together and use the fact that $L^*L=1$ to infer that, for any $\lambda ,\mu \in R_\delta$,

$$ \begin{bmatrix} 1&1\otimes E(\mu )u(\mu ) \end{bmatrix} \begin{bmatrix} 1\\ E(\lambda ) u(\lambda ) \otimes 1 \end{bmatrix} = \begin{bmatrix} 1\otimes \varphi (\mu )&1\otimes u(\mu ) \end{bmatrix} \begin{bmatrix} \varphi (\lambda )\otimes 1 \\ u(\lambda )\otimes 1 \end{bmatrix}, $$

which multiplies out to give the relation, for all $\lambda ,\mu \in R_\delta$,

$$ 1-\overline{\varphi (\mu )}\varphi (\lambda ) = \langle (1-E(\mu )^*E(\lambda ))u(\lambda ),u(\mu )\rangle _{\mathcal {N}}, $$

that is, $(\mathcal {N},u)$ is a DP-model of $\varphi$. $\square$

Let us recall the interpolation problem we posed in the Introduction.

### Definition 3.16

**The DP Pick Problem.** Given *n* distinct points $\lambda _1,\ldots ,\lambda _n$ in $R_\delta$ and $z_1,\ldots ,z_n \in \mathbb {C}$, does there exist a function $\varphi \in \textrm{H}^\infty _{\textrm{dp}}(R_\delta )$ with $\Vert \varphi \Vert _{\textrm{dp}} \le 1$ such that

3.17 $$\begin{aligned} \varphi (\lambda _j) = z_j \qquad \text {for} \ j=1,\ldots ,n ? \end{aligned}$$

We say the DP Pick Problem () is *solvable* if there exists $\varphi \in \mathcal {S}_{\textrm{dp}}$ satisfying equations (3.17).

The following theorem, which is a Pick interpolation theorem in the dp norm, is [6, Theorem 9.55].

### Theorem 3.18

Let $\delta \in (0,1)$. Let $\lambda _1, \dots , \lambda _n$ be distinct points in $R_\delta$ and let $z_1, \dots , z_n$ be arbitrary complex numbers. There exists $f \in \textrm{H}^\infty _{\textrm{dp}}(R_\delta )$ such that $\Vert f\Vert _{\textrm{dp}} \le 1$ and

$$\begin{aligned} f(\lambda _i) =z_i \;\; \text {for} \;\; i=1, \dots , n, \end{aligned}$$

if and only if there exist a pair of $n \times n$ positive semi-definite matrices $A = [ a_{ij}]$ and $B= [ b_{ij}]$ such that

$$ 1-\overline{z_i} z_j =(1-{\overline{\lambda }}_i \lambda _j)a_{ij} + (1 -\frac{\delta ^2}{{\overline{\lambda }}_i \lambda _j})b_{ij} $$

for $$i,j=1, \dots , n$$.

We also assert a dual theorem in terms of “DP Szegő kernels”, which we discuss in the next section.

## DP-Szegő kernels and normalized DP-Szegő kernels for the tuple $(\lambda _1,\dots ,\lambda _n)$

In this section we follow Abrahamse’s idea of using families of kernels to solve Pick interpolation problems. To this end we shall introduce several objects that depend on an *n*-tuple $\lambda =(\lambda _1,\dots ,\lambda _n)$ of distinct points in $R_\delta$. First we consider the set $\mathcal {F}_{\textrm{dp}}(\delta ,\lambda )$ of operators on *n*-dimensional Hilbert space with spectrum $\{\lambda _1,\dots ,\lambda _n\}$ which belong to the Douglas-Paulsen family $\mathcal {F}_{\textrm{dp}}(\delta )$. Secondly we define DP Szegő kernels for the *n*-tuple $\lambda$. We establish a close connection between these two objects in Propositions 4.9 and 4.10. Thereby, in Section 5 we shall establish a theorem analogous to Theorem 1.2, Abrahamse’s Theorem.

### Definition 4.1

We say that a kernel $k:R_\delta \times R_\delta$ is a *DP Szegő kernel on* $R_\delta$ if

4.2 $$\begin{aligned} {[}(1-{\overline{\mu }} \lambda )k(\lambda ,\mu )]\ge 0\ \ \text { and }\ \ {[}(1-\frac{\delta }{\overline{\mu }} \frac{\delta }{\lambda })k(\lambda ,\mu )] \ge 0,\qquad \text {for all} \; \lambda ,\mu \in R_\delta .\nonumber \\ \end{aligned}$$

We let

### Definition 4.3

Let $\lambda =(\lambda _1,\ldots ,\lambda _n)$ be an *n*-tuple of distinct points in $R_\delta$. We denote by $\mathcal {F}_{\textrm{dp}}(\delta ,\lambda )$ the family of operators *T* in the Douglas-Paulsen family $\mathcal {F}_{\textrm{dp}}(\delta )$ corresponding to the annulus $R_\delta$ that act on an *n*-dimensional Hilbert space $\mathcal {H}_T$ and satisfy

$$ \sigma (T)=\{\lambda _1,\ldots ,\lambda _n\}. $$

If $T\in \mathcal {F}_{\textrm{dp}}(\delta ,\lambda )$, then, as $\dim \mathcal {H}_T=n$ and $\sigma (T)$ consists of *n* distinct points, *T* is diagonalizable, that is, there exist *n* linearly independent vectors $e_1,\ldots ,e_n \in \mathcal {H}_T$ such that

4.4 $$\begin{aligned} Te_j=\lambda _j e_j \qquad \text {for} \ j=1,\ldots ,n. \end{aligned}$$

Let *g* denote the gramian of the vectors $e_1,\ldots ,e_n$, that is,

4.5 $$\begin{aligned} g=[g_{ij}], \text { where } g_{ij}=\langle e_j,e_i\rangle \qquad \text {for} \ i,j=1,\ldots ,n, \end{aligned}$$

Then, we shall prove in Proposition 4.9 that $g=[g_{ij}]$ is a positive definite $n \times n$ matrix such that

4.6 $$\begin{aligned} \left[ (1-{\overline{\lambda }}_i \lambda _j)g_{ij}\right] \ge 0\ \ \text { and }\ \ \left[ \left( 1-\frac{\delta }{\overline{\lambda _i}} \frac{\delta }{\lambda _j}\right) g_{ij}\right] \ge 0. \end{aligned}$$

### Definition 4.7

Let $\lambda _1, \dots , \lambda _n$ be *n* distinct points in $R_\delta$. We define $\mathcal {G}_{\textrm{dp}} (\lambda )$ to be the set of positive definite $n \times n$ matrices $g = [g_{ij}]$ such that

4.8 $$\begin{aligned} {[}(1-{\overline{\lambda }}_i \lambda _j)g_{ij}] \ge 0\ \ \text { and }\ \ \left[ \left( 1-\frac{\delta }{\overline{\lambda _i}} \frac{\delta }{\lambda _j}\right) g_{ij}\right] \ge 0. \end{aligned}$$

We call $g \in \mathcal {G}_{\textrm{dp}} (\lambda )$ a *DP-Szegő kernel* for the *n*-tuple $\lambda =(\lambda _1,\dots ,\lambda _n)$.

### Proposition 4.9

Let $\lambda _1, \dots , \lambda _n$ be *n* distinct points in $R_\delta$. Let $T\in \mathcal {F}_{\textrm{dp}}(\delta ,\lambda )$. Then the gramian $g=[g_{ij}]$ of vectors $e_1,\ldots ,e_n$ that satisfy the equations (4.4) and (4.5) is a positive definite $n \times n$ matrix which belongs to $\mathcal {G}_{\textrm{dp}} (\lambda )$.

### Proof

By assumption *T* is a Douglas-Paulsen operator with parameter $\delta$ that acts on an *n*-dimensional Hilbert space $\mathcal {H}_T$, *T* has *n* linearly independent eigenvectors $e_1,\dots ,e_n$ corresponding to the eigenvalues $\lambda _1,\dots ,\lambda _n$ respectively and $g_{ij} = \langle e_j,e_i\rangle$ for $i,j=1,\dots ,n$. By the definition of the Douglas-Paulsen class, $\Vert T \Vert \le 1$ and $\Vert \delta T^{-1} \Vert \le 1$, so that, for any vector $x=\sum _{j=1}^n x_j e_j$, we have

$$\begin{aligned} 0&\le \Vert x\Vert ^2 - \Vert Tx\Vert ^2 \\&= \langle \sum _{j=1}^n x_j e_j,\sum _{i=1}^n x_i e_i\rangle - \langle \sum _{j=1}^n x_j\lambda _j e_j,\sum _{i=1}^n \lambda _i x_i e_i\rangle \\&= \sum _{i,j=1}^n\overline{x_i}\left( (1-\overline{\lambda _i}\lambda _j)g_{ij}\right) x_j \\&= \begin{bmatrix} \overline{x_1}&\dots&\overline{x_n} \end{bmatrix} \begin{bmatrix} (1-\overline{\lambda _i}\lambda _j)g_{ij} \end{bmatrix} \begin{bmatrix} x_1 \\ \dots \\ x_n \end{bmatrix}. \end{aligned}$$

Thus

$$ \big [(1-{\overline{\lambda }}_i\lambda _j)g_{ij}\big ]_{i,j=1}^n \ge 0. \ $$

Likewise, the relation $\Vert \delta T^{-1}x\Vert \le \Vert x\Vert$ holds for any vector $x=\sum _{j=1}^n x_j e_j \in \mathcal {H}_T$. Therefore, we have

$$\begin{aligned} 0&\le \Vert x\Vert ^2 - \left\| \delta T^{-1}x\right\| ^2 \\&= \langle \sum _{j=1}^n x_j e_j,\sum _{i=1}^n x_i e_i\rangle - \langle \sum _{j=1}^n \frac{\delta }{\lambda _j} x_j e_j , \sum _{i=1}^n \frac{\delta }{\lambda _i} x_i e_i \rangle \\&= \sum _{i,j=1}^n\overline{x_i}\left( \left( 1-\frac{\delta }{\overline{\lambda _i}}\frac{\delta }{\lambda _j}\right) g_{ij}\right) x_j \\&= \begin{bmatrix} \overline{x_1}&\dots&\overline{x_n} \end{bmatrix} \begin{bmatrix} (1-{\frac{\delta }{\overline{\lambda _i}}} \frac{\delta }{\lambda _j}) g_{ij} \end{bmatrix} \begin{bmatrix} x_1 \\ \dots \\ x_n \end{bmatrix}. \end{aligned}$$

Thus

$$ \big [\left( 1-{\frac{\delta }{\overline{\lambda _i}}} \frac{\delta }{\lambda _j}\right) g_{ij}\big ]_{i,j=1}^n \ge 0. $$

Therefore, $g=[g_{ij}]$ is a positive definite DP-Szegő kernel for the *n*-tuple $\lambda =(\lambda _1,\dots ,\lambda _n)$. $\square$

Let $g \in \mathcal {G}_{\textrm{dp}} (\lambda )$, so that $g > 0$. By Moore’s theorem [6, Theorem 2.5], *g* is the gramian matrix of a basis $e_1, \dots , e_n$ of an *n*-dimensional Hilbert space ${{\mathcal {H}}}$.

### Proposition 4.10

Let $\lambda _1, \dots , \lambda _n$ be *n* distinct points in $R_\delta$. Let $g \in \mathcal {G}_{\textrm{dp}} (\lambda )$. Let *g* be the gramian matrix of a basis $e_1, \dots , e_n$ of an *n*-dimensional Hilbert space ${{\mathcal {H}}}$. Define $T \in \mathcal {B}(\mathcal {H})$ by

4.11 $$\begin{aligned} T e_j= \lambda _j e_j, \ j =1, \dots , n. \end{aligned}$$

Then $$T \in \mathcal {F}_{\textrm{dp}}(\delta ,\lambda )$$.

### Proof

Let us show that *T* is a Douglas-Paulsen operator. If $x= \sum _{j=1}^n \xi _j e_j \in \mathcal {H}$, $Tx = \sum _{j=1}^n \xi _j \lambda _j e_j$ and

4.12 $$\begin{aligned} \Vert Tx\Vert ^2&= \big \langle \, \sum _{j=1}^n \xi _j \lambda _j e_j , \sum _{i=1}^n \xi _i \lambda _i e_i\, \big \rangle \nonumber \\&=\sum _{j,i=1}^n \xi _j \lambda _j {\overline{\xi }}_i {\overline{\lambda }}_i \langle \, e_j,e_i \,\rangle \nonumber \\&=\sum _{j,i=1}^n {\overline{\lambda }}_i \lambda _j \xi _j {\overline{\xi }}_i \, g_{ij}. \end{aligned}$$

Hence

$$ \Vert x\Vert ^2 - \Vert Tx\Vert ^2 =\sum _{j,i=1}^n (1 - {\overline{\lambda }}_i \lambda _j) \, g_{ij} \xi _j {\overline{\xi }}_i . $$

By hypothesis,

$$\begin{aligned} {[}(1-{\overline{\lambda }}_i \lambda _j)g_{ij}] \ge 0, \end{aligned}$$

and so $\Vert x\Vert ^2 - \Vert Tx\Vert ^2 \ge 0$. Thus $\Vert T\Vert \le 1$. Similarly, using the hypothesis

$$ \left[ \left( 1-\frac{\delta }{\overline{\lambda _i}} \frac{\delta }{\lambda _j}\right) g_{ij}\right] \ge 0, $$

one can show that $\Vert \delta T^{-1}\Vert \le 1$. Therefore *T* is a Douglas-Paulsen operator. In addition, by the definition (4.11) of *T* ,

$$ \sigma (T)=\{\lambda _1,\ldots ,\lambda _n\} \subset R_\delta . $$

thus $$T \in \mathcal {F}_{\textrm{dp}}(\delta ,\lambda )$$. $$\square $$

### Proposition 4.13

Let $\lambda _1,\ldots ,\lambda _n$ be *n* distinct points in $R_\delta$ and $z_1,\ldots ,z_n \in \mathbb {C}$. If the DP Pick Problem 3.16 is solvable, then, for any positive definite $g \in \mathcal {G}_{\textrm{dp}} (\lambda )$,

4.14 $$\begin{aligned} {[}(1-{\overline{z}}_i z_j)g_{ij}] \ge 0. \end{aligned}$$

### Proof

By assumption, the DP Pick Problem 3.16 is solvable, that is, there exist a function $\varphi \in \textrm{H}^\infty _{\textrm{dp}}(R_\delta )$ with $\Vert \varphi \Vert _{\textrm{dp}} \le 1$ and satisfying

4.15 $$\begin{aligned} \varphi (\lambda _j) = z_j,\qquad j=1,\ldots ,n. \end{aligned}$$

Let $g \in \mathcal {G}_{\textrm{dp}} (\lambda )$, and so $g > 0$. By Moore’s theorem [6, Theorem 2.5], *g* is the gramian matrix of a basis $e_1, \dots , e_n$ of an *n*-dimensional Hilbert space ${{\mathcal {H}}}$. Define $T \in \mathcal {B}(\mathcal {H})$ by

4.16 $$\begin{aligned} T e_j= \lambda _j e_j, \ j =1, \dots , n. \end{aligned}$$

By Proposition 4.10, $T \in \mathcal {F}_{\textrm{dp}}(\delta ,\lambda )$. By assumption, $\varphi \in \mathcal {S}_{\textrm{dp}}$, and so $\Vert \varphi (T)\Vert \le 1$. For any $x= \sum _{j=1}^n \xi _j e_j \in \mathcal {H}$,

4.17 $$\begin{aligned} \varphi (T) x&= \varphi (T)\sum _{j=1}^n \xi _j e_j \nonumber \\&=\sum _{j=1}^n \xi _j \varphi (\lambda _j) e_j = \sum _{j=1}^n \xi _j z_j e_j. \end{aligned}$$

Therefore, by equation (4.17), the condition $\Vert \varphi (T)\Vert \le 1$ translates into

$$ {[}(1-{\overline{z}}_i z_j)g_{ij}] \ge 0. $$

$$\square $$

### Definition 4.18

Let $\lambda _1, \dots , \lambda _n$ be *n* distinct points in $R_\delta$. We say that a DP-Szegő kernel $[g_{ij}] \in \mathcal {G}_{\textrm{dp}} (\lambda )$ is *normalized* if $g_{ii}=1$ for $i=1,\dots ,n$.

Let $\mathcal {G}_{\textrm{dp}}^{\textrm{norm}} (\lambda )$ denote the set of normalized DP-Szegő kernels for the *n*-tuple $(\lambda _1,\dots ,\lambda _n)$.

### Remark 4.19

Let $\lambda _1, \dots , \lambda _n$ be *n* distinct points in $R_\delta$. Every DP-Szegő kernel $[g_{ij}]$ from $\mathcal {G}_{\textrm{dp}} (\lambda )$ is diagonally congruent to a normalized DP-Szegő kernel.

### Proof

For any matrix $[g_{ij}]\in \mathcal {G}_{\textrm{dp}} (\lambda )$, we can define a positive definite matrix $[h_{ij}]$ by

4.20 $$\begin{aligned} h_{ii}=1\ \text {for}\ i=1,\dots ,n \ \text {and}\ h_{ij}= c_i^{-1}g_{ij} c_j^{-1}\ \text {if}\ i\ne j \end{aligned}$$

where

4.21 $$\begin{aligned} c_i = \sqrt{g_{ii}}\ \text {if}\ g_{ii}\ne 0\ \text {and} \ c_i=1\ \text {if}\ g_{ii}=0. \end{aligned}$$

Then $h_{ii}=1$ for each *i*, and

4.22 $$\begin{aligned} \begin{bmatrix} h_{ij} \end{bmatrix}_{i,j=1}^n = C^*\begin{bmatrix} g_{ij} \end{bmatrix}_{i,j=1}^n C \ \text {where}\ C= \textrm{diag} \{1/c_1, \dots , 1/c_n\}. \end{aligned}$$

On conjugating the inequalities (4.8) by the matrix *C* we find that $[h_{ij}]$ belongs to $\mathcal {G}_{\textrm{dp}}^{\textrm{norm}} (\lambda )$. $\square$

### Proposition 4.23

Let $\lambda _1, \dots , \lambda _n$ be *n* distinct points in $R_\delta$. The set $\mathcal {G}_{\textrm{dp}}^{\textrm{norm}} (\lambda )$ is compact in the topology of the space of $n\times n$ complex matrices. Moreover, for fixed target data $z_1,\dots ,z_n$,

4.24 $$\begin{aligned} \begin{bmatrix} (1-{\overline{z}}_i z_j) g_{ij} \end{bmatrix}_{i,j=1}^n \ge 0\ \text {for all}\ g\in \mathcal {G}_{\textrm{dp}} (\lambda ) \end{aligned}$$

if and only if

4.25 $$\begin{aligned} \begin{bmatrix} (1-{\overline{z}}_i z_j) g_{ij} \end{bmatrix}_{i,j=1}^n \ge 0\ \text {for all}\ g\in \mathcal {G}_{\textrm{dp}}^{\textrm{norm}} (\lambda ). \end{aligned}$$

### Proof

Consider any matrix $g=[g_{ij}] \in \mathcal {G}_{\textrm{dp}}^{\textrm{norm}} (\lambda )$. Since *g* is positive definite, the principal minor on rows *i* and *j* is non-negative, which is to say that $1-|g_{ij}|^2 \ge 0$ for $i,j=1,\dots ,n$. It follows that the operator norm $\Vert g\Vert \le n$, and so $\mathcal {G}_{\textrm{dp}}^{\textrm{norm}} (\lambda )$ is bounded. Let us prove that $\mathcal {G}_{\textrm{dp}}^{\textrm{norm}} (\lambda )$ is sequentially compact.

Let $g^\ell$, $\ell =1,2, \dots$, be a sequence in $\mathcal {G}_{\textrm{dp}}^{\textrm{norm}} (\lambda )$. We claim that $(g^\ell )_{\ell \ge 1}$ has a subsequence that converges to an element of $\mathcal {G}_{\textrm{dp}}^{\textrm{norm}} (\lambda )$. For each $\ell$, since $g^\ell$ is non-singular, by the definition of $\mathcal {G}_{\textrm{dp}}^{\textrm{norm}} (\lambda )$, we may pick a basis $e_1^\ell , \dots , e_n^\ell$ of $\mathbb {C}^n$ such that $g^\ell$ is the gramian matrix of the basis $e_1^\ell , \dots , e_n^\ell$, which is to say that

4.26 $$\begin{aligned} g^\ell =[g_{ij}^\ell ], \text { where } g_{ij}^\ell =\langle e_j^\ell ,e_i^\ell \rangle \qquad \text {for} \ i,j=1,\ldots ,n. \end{aligned}$$

Define $$T^\ell \in \mathcal {B}(\mathbb {C}^n)$$ by

4.27 $$\begin{aligned} T^\ell e_j^\ell = \lambda _j e_j^\ell , \ j =1, \dots , n. \end{aligned}$$

Note that since $g^\ell$ is normalised, that is,

$$ g_{ii}^\ell =\langle e_i^\ell ,e_i^\ell \rangle = 1 \qquad \text {for} \ i=1,\ldots ,n, $$

and so $\Vert e_i^\ell \Vert = 1$ for $i=1,\ldots ,n$. Then, by Proposition 4.10,

$$ \sigma (T^\ell )=\{\lambda _1,\ldots ,\lambda _n\} $$

and $T^\ell \in \mathcal {F}_{\textrm{dp}}(\delta ,\lambda )$. By the compactness of the unit sphere in $\mathbb {C}^n$, we can choose a subsequence $(e_i^{\ell _k})_{k \ge 1}$ of $(e_i^{\ell })_{\ell \ge 1}$ such that $(e_j^{\ell _k})$ converges to a unit vector $v_j \in \mathbb {C}^n$ as $k \rightarrow \infty$ for $j=1,\dots ,n$. By the compactness of the unit ball in $\mathcal {B}(\mathbb {C}^n)$, by passing to a further subsequence $(e^{\ell _k})_{k\ge 1}$ of $(e^\ell )_{\ell \ge 1}$ we can arrange also that $(T^{\ell _k})$ converges to a limit $T \in \mathcal {B}(\mathbb {C}^n)$ as $k \rightarrow \infty$. In the relations

4.28 $$\begin{aligned} T^{\ell _k} e_j^{\ell _k} = \lambda _j e_j^{\ell _k}, \ j =1, \dots , n, \end{aligned}$$

let $k \rightarrow \infty$ to obtain

4.29 $$\begin{aligned} T v_j = \lambda _j v_j \qquad \text {and } \ \Vert v_j \Vert = 1\ \qquad j =1, \dots , n. \end{aligned}$$

Thus

$$ \sigma (T^\ell )=\{\lambda _1,\ldots ,\lambda _n\}, $$

the eigenvectors $v_1,\dots ,v_n$ of *T* corresponding to the distinct eigenvalues $\lambda _1,\ldots ,\lambda _n$ are linearly independent and therefore span $\mathbb {C}^n$, and $T^\ell \in \mathcal {F}_{\textrm{dp}}(\delta ,\lambda )$. Let *g* be the Gramian of the vectors $v_1, \dots , v_n$ in $\mathbb {C}^n$: then *g* is positive definite, and by Proposition 4.9, $g\in \mathcal {G}_{\textrm{dp}}^{\textrm{norm}} (\lambda )$. We have

$$ g_{ij}=\langle v_j,v_i\rangle = \lim _{k \rightarrow \infty } \langle v_j^{\ell _k},v_i^{\ell _k}\rangle = \lim _{k\rightarrow \infty } g_{ij}^{\ell _k} $$

for $i,j=1,\dots ,n$, and so $g^{\ell _k} \rightarrow g$ as $k\rightarrow \infty$. We have shown that $\mathcal {G}_{\textrm{dp}}^{\textrm{norm}} (\lambda )$ is sequentially compact in the metrizable topology of $\mathcal {B}(\mathbb {C}^n)$, hence it is compact.

To prove the “Moreover”, fix target data $z_1,\dots ,z_n$. Since $\mathcal {G}_{\textrm{dp}} (\lambda ) \supset \mathcal {G}_{\textrm{dp}}^{\textrm{norm}} (\lambda )$, trivially statement (4.24) implies statement (4.25). Conversely, suppose statement (4.25) holds and consider any kernel $g \in \mathcal {G}_{\textrm{dp}} (\lambda )$. Define matrices $h=[h_{ij}]$ and *C* by the relations (4.20), (4.21) and (4.22). Then $h \in \mathcal {G}_{\textrm{dp}}^{\textrm{norm}} (\lambda )$, and so, by assumption,

4.30 $$\begin{aligned} {[}(1-{\overline{z}}_i z_j)h_{ij}] \ge 0 \end{aligned}$$

Conjugate this matrix inequality by $\textrm{diag}\{c_1,\dots ,c_n\}$ to obtain the relation (4.24). Thus the relation (4.25) implies the relation (4.24). $\square$

Say that a DP-Szegő kernel *g* on $R_\delta$ is *reducible* if there exist DP-Szegő kernels *h* and *k* on $R_\delta$ such that $g=h+k$ and neither *h* nor *k* is diagonally congruent to *g*. Here two kernels *g* and *h* on $R_\delta$ are said to be diagonally congruent if there exists a function $c:R_\delta \rightarrow \mathbb {C}\setminus \{0\}$ such that, for all $\lambda ,\mu \in R_\delta$, $h(\lambda ,\mu )= c(\lambda )g(\lambda ,\mu ) \overline{c(\mu )}$. A DP-Szegő kernel is *irreducible* if it is not reducible. Clearly, if DP Pick data $\lambda _j \mapsto z_j, j=1,\dots ,n$, are such that

$$ {[}(1-{\overline{z}}_iz_j)g_{ij}] \ge 0\ \text {and}\ {[}1-\frac{\delta ^2}{\overline{z_i}z_j} g_{ij}] \ge 0 $$

for all irreducible DP Szegő kernels *g* then the same inequality holds for *all* DP Szegő kernels, and consequently the DP pick interpolation problem is solvable. Since the class of irreducible DP Szegő kernels is likely to be *much* smaller than the class of all DP Szegő kernels, it would be valuable to identify the irreducible DP Szegő kernels on $R_\delta$.

### Problem 4.31

Find an effective description of the irreducible DP Szegő kernels on $R_\delta$.

## The DP Pick problem and DP-Szegő kernels

In this section we shall prove our main theorem, which is a solvability criterion for DP Pick problems in terms of DP-Szegő kernels. We also present some examples which illustrate the relationship between the Pick and DP Pick interpolation problems.

The following notation and terminology will be needed in the proofs.

### Definition 5.1

Let $H_n$ be the real linear space of Hermitian matrices in $\mathbb {C}^{n \times n}$. A subset *P* of $H_n$ is called a *cone* if the following conditions are satisfied: (i) $P +P \subseteq P$, (ii) $P \cap (-P) = \{0\}$ and (iii) $\alpha P \subseteq P$ whenever $\alpha \in \mathbb {R}$ and $\alpha \ge 0$.

### Theorem 5.2

Let $\lambda _1,\dots ,\lambda _n \in R_\delta$ be distinct and let $z_1,\dots ,z_n\in \mathbb {C}$. There exists $\varphi \in \mathcal {S}_{\textrm{dp}}$ such that

$$\begin{aligned} \varphi (\lambda _j) = z_j \qquad \text {for} \ j=1,\ldots ,n, \end{aligned}$$

if and only if, for all $g \in \mathcal {G}_{\textrm{dp}} (\lambda )$,

5.3 $$\begin{aligned} {[}(1-{\overline{z}}_i z_j)g_{ij}] \ge 0. \end{aligned}$$

### Proof

Implication $\Rightarrow$ follows from Proposition 4.13.

To prove $\Leftarrow$, suppose that

5.4 $$\begin{aligned} {[}(1-{\overline{z}}_i z_j)g_{ij}] \ge 0 \end{aligned}$$

for all $$g \in \mathcal {G}_{\textrm{dp}} (\lambda )$$.

By Theorem 3.18, to show that the DP Pick Problem () is solvable it suffices to prove that there exist a pair of $n \times n$ positive semi-definite matrices $A = [ a_{ij}]$ and $B= [ b_{ij}]$ such that

$$ 1-\overline{z_i} z_j =(1-\overline{\lambda _i} \lambda _j)a_{ij} + ({\overline{\lambda }}_i \lambda _j-\delta ^2)b_{ij} $$

for all $i,j=1, \dots , n$. Let $H_n$ be the real linear space of Hermitian matrices in $\mathbb {C}^{n \times n}$, and let

5.5 $$\begin{aligned} {{\mathcal {C}}}  &   = \left\{ [(1-\overline{\lambda _i} \lambda _j) a_{ij} ]_{i,j=1}^n + \left[ \left( 1-\frac{\delta ^2}{\overline{\lambda _i} \lambda _j} \right) b_{ij} \right] _{i,j=1}^n: [a_{ij}]_{i,j=1}^n \right. \nonumber \\  &   \left. \ge 0 \ \text {and} \ [b_{ij}]_{i,j=1}^n \ge 0 \right\} . \end{aligned}$$

The subset ${{\mathcal {C}}}$ is a closed convex cone in $H_n$.

Note that every $n \times n$ positive semi-definite matrix $[a_{ij}]_{i,j=1}^n$ belongs to ${{\mathcal {C}}}$. By the positivity of Szegő kernel $\left[ \frac{1}{1- \overline{\lambda _i} \lambda _j} \right] _{i,j=1}^n$, the $n \times n$ matrix of the form

$$ \left[ \frac{a_{ij}}{1- \overline{\lambda _i} \lambda _j} \right] _{i,j=1}^n $$

is also positive semi-definite. In the definition of ${{\mathcal {C}}}$ (5.5) we may replace $[a_{ij}]_{i,j=1}^n$ by $\left[ \frac{a_{ij}}{1- {\overline{\lambda }}_i \lambda _j} \right] _{i,j=1}^n$ and $[b_{ij}]_{i,j=1}^n$ by the zero matrix, to deduce that $[a_{ij}]_{i,j=1}^n$ belongs to ${{\mathcal {C}}}$.

By the Hahn-Banach theorem, to show that $[1-\overline{z_i} z_j]_{i,j=1}^n$ belongs to ${{\mathcal {C}}}$ it suffices to prove that, for every real linear functional ${{\mathcal {L}}}$ on $H_n$, ${{\mathcal {L}}} \ge 0$ on ${{\mathcal {C}}}$ implies ${{\mathcal {L}}} ( [1-\overline{z_i} z_j]_{i,j=1}^n ) \ge 0$.

Extend ${\mathcal {L}}$ to a complex linear functional ${\widetilde{\mathcal {L}}}$ on ${\mathbb {C}}^{n \times n}$ by

$$ \widetilde{{\mathcal {L}}}(X + {\textrm{i}} \, Y) = {{\mathcal {L}}}(X) + {\mathrm i} \, {{\mathcal {L}}}(Y) $$

for $X, Y \in H_n$. Now define a pre-inner product $\langle \cdot , \cdot \rangle _L$ on $\mathbb {C}^n$ by

$$ \langle \, c, d\,\rangle _L = \widetilde{{\mathcal {L}}} (c \otimes d) $$

for $c, d \in \mathbb {C}^n$. Here $c \otimes d \in \mathbb {C}^{n \times n}$, defined by

$$ (c \otimes d)(x) = \langle \, x, d \,\rangle _{\mathbb {C}^n} c \; \; \; \text {for all} \ x \in \mathbb {C}^n. $$

Note that, for any $c \in \mathbb {C}^n$,

$$ \langle \, c, c\,\rangle _L = \widetilde{{\mathcal {L}}} (c \otimes c) = {{\mathcal {L}}} (c \otimes c) \ge 0. $$

Let

$$ {{\mathcal {N}}}= \{ x \in \mathbb {C}^n: \, \langle \, x, x\,\rangle _L = 0\}. $$

Then ${{\mathcal {N}}}$ is a subspace of $\mathbb {C}^n$, and $\langle \cdot , \cdot \rangle _L$ induces an inner product on $\mathbb {C}^n /{{\mathcal {N}}}$.

Let $e_1, \dots , e_n$ be the standard basis of $\mathbb {C}^n$ and let $T \in \mathcal {B}(\mathbb {C}^n)$ defined by

5.6 $$\begin{aligned} T e_j= \lambda _j e_j, \ j =1, \dots , n. \end{aligned}$$

Let us construct an operator ${\widetilde{T}}$ on $\mathbb {C}^n/{{\mathcal {N}}}$ such that $\Vert {\widetilde{T}}\Vert \le 1$ and $\Vert \delta {\widetilde{T}}^{-1}\Vert \le 1$. For $x= \sum _{j=1}^n \xi _j e_j \in \mathbb {C}^n$, we have

5.7 $$\begin{aligned} \langle \, x, x\,\rangle _L - \langle \, Tx, Tx\,\rangle _L  &   = \widetilde{{\mathcal {L}}} (x \otimes x) - \widetilde{{\mathcal {L}}} (Tx \otimes Tx) \nonumber \\  &   =\widetilde{{\mathcal {L}}}\left( \sum _{j=1}^n \xi _j e_j\,\otimes \sum _{i=1}^n \xi _i e_i \right) - \widetilde{{\mathcal {L}}}\left( \sum _{j=1}^n \xi _j \lambda _j e_j\,\otimes \sum _{i=1}^n \xi _i \lambda _i e_i \right) \nonumber \\  &   =\widetilde{{\mathcal {L}}}\left( \sum _{j,i=1}^n \xi _j \overline{\xi }_i e_j\,\otimes e_i \right) - \widetilde{{\mathcal {L}}}\left( \sum _{j,i=1}^n \xi _j \lambda _j {\overline{\xi }}_i {\overline{\lambda }}_i e_j\,\otimes e_i \right) \nonumber \\  &   =\widetilde{{\mathcal {L}}}\left( \sum _{j,i=1}^n (1- {\overline{\lambda }}_i \lambda _j ) {\overline{\xi }}_i \xi _j e_j\,\otimes e_i \right) \nonumber \\  &   =\widetilde{{\mathcal {L}}} \left[ (1- {\overline{\lambda }}_i \lambda _j ) {\overline{\xi }}_i \xi _j \right] _{j,i=1}^n \nonumber \\  &   ={{\mathcal {L}}} \left[ (1- {\overline{\lambda }}_i \lambda _j ) \overline{\xi }_i \xi _j \right] _{j,i=1}^n \ge 0 \;\;\; \text{ since }\;\;\mathcal { L} \ge 0 \ \text {on} \ {{\mathcal {C}}}. \end{aligned}$$

Thus

5.8 $$\begin{aligned} \langle \, x, x\,\rangle _L - \langle \, Tx, Tx\,\rangle _L \ge 0, \end{aligned}$$

and so $x \in {{\mathcal {N}}}$ implies that $Tx \in {{\mathcal {N}}}$. Hence *T* induces an operator ${\widetilde{T}}$ on $\mathbb {C}^n /{\mathcal N}$ by

$$ \widetilde{T} (x+{{\mathcal {N}}}) = T x + {{\mathcal {N}}}, $$

and $\Vert \widetilde{T} (x+{{\mathcal {N}}}) \Vert ^2 \le \Vert x+{{\mathcal {N}}} \Vert ^2$ for all $(x+{{\mathcal {N}}}) \in \mathbb {C}^n /{{\mathcal {N}}}$, which implies that

5.9 $$\begin{aligned} \Vert {\widetilde{T}}\Vert \le 1. \end{aligned}$$

Notice that $\sigma (T)=\{\lambda _1,\dots ,\lambda _n\} \subset R_\delta$ and so *T* is invertible. Moreover

$$ \delta T^{-1}e_j = \frac{\delta }{\lambda _j} e_j \ \text {for} \ j=1,\dots ,n, $$

and so, in the chain of equations leading to equation (5.7), we may replace *T* by $\delta T^{-1}$ and $\lambda _j$ by $\frac{\delta }{\lambda _j}$ to deduce that, for $x=\sum _{j=1}^n \xi _j e_j \in \mathbb {C}^n$,

$$ \langle x,x\rangle _L-\langle \delta T^{-1}x,\delta T^{-1}x\rangle _L = \mathcal {L}\left[ \left( 1-\tfrac{\delta }{\overline{\lambda _i}} \tfrac{\delta }{\lambda _j}\right) \overline{\xi _i}\xi _j\right] _{j,i=1}^n. $$

Clearly $\left[ \left( 1-\tfrac{\delta }{\overline{\lambda _i}} \tfrac{\delta }{\lambda _j}\right) \overline{\xi _i}\xi _j\right] _{j,i=1}^n \in \mathcal {C}$ (take $a_{ij}=0, b_{ij}=\overline{\xi _i}\xi _j$ in the defining expression (5.5)), and so, since $\mathcal {L} \ge 0$ on $\mathcal {C}$, we have

5.10 $$\begin{aligned} \langle x,x\rangle _L-\langle \delta T^{-1}x,\delta T^{-1}x\rangle _L \ge 0. \end{aligned}$$

Thus $x \in {{\mathcal {N}}}$ implies that $\delta T^{-1}x \in {\mathcal N}$, and therefore $\delta T^{-1}$ induces an operator $\widetilde{(\delta T^{-1})}$ on $\mathbb {C}^n /\mathcal {N}$ by

$$ \widetilde{(\delta T^{-1})} (x+\mathcal {N}) = \delta T^{-1}x + \mathcal {N}, $$

and in the light of inequality (5.10),

5.11 $$\begin{aligned} \Vert \widetilde{(\delta T^{-1})}\Vert \le 1. \end{aligned}$$

We have, for any $x\in \mathbb {C}^n$,

$$ {\widetilde{T}} \widetilde{(\delta T^{-1})} (x+\mathcal {N}) = {\widetilde{T}} (\delta T^{-1}x+\mathcal {N}) = T \delta T^{-1}x+\mathcal {N} = \delta (x+\mathcal {N}), $$

and so $\widetilde{(\delta T^{-1})} = \delta ({\widetilde{T}})^{-1}$. Hence, by the inequality (5.11),

$$ \Vert \delta ({\widetilde{T}})^{-1}\Vert = \Vert \widetilde{(\delta T^{-1})} \Vert \le 1. $$

Therefore, ${\tilde{T}}$ is a Douglas-Paulsen operator. Since the eigenvalues of *T*, which are $\lambda _1, \dots , \lambda _n$, belong to $R_\delta$, $\sigma (T) \subseteq R_\delta$, and so the operator *T* belongs to $\mathcal {F}_{\textrm{dp}}(\delta ,\lambda )$. Therefore, by Proposition 4.9, $[ \langle \, e_j,e_i \,\rangle _L]_{i,j=1}^n$ belongs to $\mathcal {G}_{\textrm{dp}} (\lambda )$.

Let $g_{ij}=\langle \, e_j,e_i \,\rangle _L$ for $i,j=1,\dots ,n$. By supposition (5.4),

$$ {[}(1-{\overline{z}}_i z_j) \langle \, e_j,e_i \,\rangle _L]_{i,j=1}^n \ge 0.$$

Choose a polynomial *p* such that $p(\lambda _i) = z_i$, $i =1,\dots , n$. Then $~p({\widetilde{T}})e_i = z_i e_i$, $i =1,\dots , n$. Observe that

$$\begin{aligned} \left[ \langle (1-p({\tilde{T}})^*p({\tilde{T}}))e_j,e_i\rangle \right]&= \left[ \langle e_j,e_i\rangle - \langle p({\tilde{T}})e_j,p({\tilde{T}})e_i\rangle \right] \\&= \left[ \langle e_j,e_i\rangle - \langle z_je_j,z_ie_i\rangle \right] \\&= \left[ (1- {\overline{z}}_i z_j)g_{ij} \right] \ge 0. \end{aligned}$$

Therefore, $\Vert p({\widetilde{T}})\Vert \le 1$. Choose $$c = \begin{bmatrix} 1 \\ \dots \\ 1 \end{bmatrix} \in \mathbb {C}^n$$. Then

$$\begin{aligned} \langle \,( 1- p({\widetilde{T}})^* p({\widetilde{T}})) c, c\,\rangle _L \ge 0, \end{aligned}$$

that is,

$$ {{\mathcal {L}}} ( [1-{\overline{z}}_i z_j]_{i,j=1}^n *c c^*) \ge 0, \ \text {and so } \ {{\mathcal {L}}} ( [1-{\overline{z}}_i z_j]_{i,j=1}^n ) \ge 0, $$

where $*$ denotes the Schur product of matrices.

Thus, for every real linear functional ${{\mathcal {L}}}$ on $H_n$ such that ${{\mathcal {L}}} \ge 0$ on ${{\mathcal {C}}}$ we have

$${{\mathcal {L}}} ( [1-{\overline{z}}_i z_j]_{i,j=1}^n ) \ge 0. $$

Hence $[1-\overline{z}_i z_j]_{i,j=1}^n$ belongs to ${{\mathcal {C}}}$. $\square$

We show in the next theorem that, as in the classical Pick theorem, if a DP Pick problem is solvable then it is solvable by a *rational* function in $\mathcal {S}_{\textrm{dp}}$.

### Theorem 5.12

Let $\lambda _1,\dots ,\lambda _n \in R_\delta$ be distinct and let $z_1,\dots ,z_n\in \mathbb {C}$. If the DP Pick problem

$$ \lambda _j \mapsto z_j \quad \text {for} \ j=1,\ldots ,n $$

is solvable, then there exists a rational function $\varphi \in \mathcal {S}_{\textrm{dp}}$ which satisfies the equations

5.13 $$\begin{aligned} \varphi (\lambda _j) = z_j \qquad \text {for} \ j=1,\ldots ,n, \end{aligned}$$

and has a model $(\mathcal {M},u)$, with $u:R_\delta \rightarrow \mathcal {M}$ holomorphic, so that

5.14 $$\begin{aligned} 1-\overline{\varphi (\mu )}\varphi (\lambda ) = \Big \langle \, \big (1-E(\mu )^*E(\lambda )\big )\ u(\lambda )\, ,u(\mu )\, \Big \rangle _\mathcal {M}\ \text {for}\ \lambda ,\mu \in R_\delta , \end{aligned}$$

where $\mathcal {M}$ can be written as $\mathcal {M}=\mathcal {M}_1\oplus \mathcal {M}_2$, $\dim \mathcal {M}\le 2n$ and $E:R_\delta \rightarrow \mathcal {B}(\mathcal {M})$ is defined by the formula

5.15 $$\begin{aligned} E(\lambda )=\begin{bmatrix}\lambda &  0\\ 0 &  \frac{\delta }{\lambda }\end{bmatrix}, \ \qquad \text {for}\ \lambda \in R_\delta , \end{aligned}$$

with respect to this orthogonal decomposition of $\mathcal {M}$.

### Proof

Suppose that

$$ \lambda _j \mapsto z_j\ \text {for}\ j=1,\dots ,n $$

is a solvable DP-Pick problem. By Theorem 3.18, there exist positive semi-definite $n\times n$ matrices $$a=\begin{bmatrix} a_{ij} \end{bmatrix}$$ and $$b=\begin{bmatrix} b_{ij} \end{bmatrix}$$ such that

5.16 $$\begin{aligned} 1-{\overline{z}}_i z_j = (1-{\overline{\lambda }}_i\lambda _j) a_{ij} + (1- \frac{\delta ^2}{\overline{\lambda _i}\lambda _j}) b_{ij} \quad \text {for} \ i,j=1,\dots ,n. \end{aligned}$$

Let the ranks of the matrices *a*, *b* be $r_1,r_2$ respectively, so that $r_1\le n, r_2\le n$. Then there exist vectors $x_1,\dots ,x_n \in \mathbb {C}^{r_1}, y_1,\dots ,y_n\in \mathbb {C}^{r_2}$ such that

$$ a_{ij}= \langle x_j,x_i\rangle _{\mathbb {C}^{r_1}}\ \text {and}\ b_{ij}= \langle y_j,y_i\rangle _{\mathbb {C}^{r_2}} \quad \text {for}\ i,j=1,\dots ,n. $$

Substituting these relations into the equations (5.16) and re-arranging, we obtain the relations

$$\begin{aligned} 1+\langle \lambda _j x_j,\lambda _i x_i\rangle _{\mathbb {C}^{r_1}} +\langle \frac{\delta }{\lambda _j}y_j,\frac{\delta }{\lambda _i}y_i\rangle _{\mathbb {C}^{r_2}}&= {\overline{z}}_i z_j + \langle x_j,x_i\rangle _{\mathbb {C}^{r_1}} +\langle y_j,y_i\rangle _{\mathbb {C}^{r_2}}\ \\  &\quad \text {for}\ i,j=1,\dots ,n. \end{aligned}$$

These equations can in turn be expressed by saying that the families of vectors

$$ \begin{pmatrix} 1 \\ \lambda _j x_j \\ \frac{\delta }{\lambda _j} y_j \end{pmatrix}_{j=1,\dots ,n}\ \text {and}\ \begin{pmatrix} z_j \\ x_j \\ y_j \end{pmatrix}_{j=1,\dots ,n} $$

in $\mathbb {C}\oplus \mathbb {C}^{r_1}\oplus \mathbb {C}^{r_2}$ have the same gramians. It follows from the “lurking isometry lemma” [6, Lemma 2.18] that there exists an isometry $L\in \mathcal {B}(\mathbb {C}\oplus \mathbb {C}^{r_1}\oplus \mathbb {C}^{r_2})$ such that

5.17 $$\begin{aligned} L\begin{pmatrix} 1 \\ \lambda _j x_j \\ \frac{\delta }{\lambda _j} y_j \end{pmatrix} = \begin{pmatrix} z_j \\ x_j \\ y_j \end{pmatrix}\ \text {for}\ j=1,\dots ,n. \end{aligned}$$

Express *L* by an operator matrix with respect to the orthogonal decomposition $\mathbb {C}\oplus \left( \mathbb {C}^{r_1} \oplus \mathbb {C}^{r_2}\right)$:

$$ L \sim \begin{bmatrix} a &  1\otimes \beta \\ \gamma \otimes 1 & D \end{bmatrix}, $$

where $a\in \mathbb {C},\ \beta ,\gamma \in \mathbb {C}^{r_1} \oplus \mathbb {C}^{r_2}$ and $D \in \mathcal {B}(\mathbb {C}^{r_1} \oplus \mathbb {C}^{r_2})$. In terms of these variables and our previous notation

$$ E(\lambda ) {\mathop {=}\limits ^\textrm{def}}\begin{bmatrix} \lambda &  0\\ 0 &  \frac{\delta }{\lambda } \end{bmatrix} :\mathbb {C}^{r_1} \oplus \mathbb {C}^{r_2} \rightarrow \mathbb {C}^{r_1} \oplus \mathbb {C}^{r_2} \ \text {for}\ \lambda \in R_\delta , $$

equation (5.17) can be written

5.18 $$\begin{aligned} a+ \langle E(\lambda _j)\begin{pmatrix} x_j \\ y_j\end{pmatrix},\beta \rangle _{\mathbb {C}^{r_1} \oplus \mathbb {C}^{r_2}}&= z_j \nonumber \\ \gamma + DE(\lambda _j)\begin{pmatrix} x_j \\ y_j\end{pmatrix}&= \begin{pmatrix} x_j \\ y_j\end{pmatrix} \end{aligned}$$

for $j=1,\dots ,n$. Observe that, for any $\lambda \in R_\delta$, $\Vert E(\lambda )\Vert < 1$. As also $\Vert D\Vert \le 1$ (since *L* is an isometry), $1-DE(\lambda _j)$ is invertible for each *j*. The equations (5.18) can therefore be solved to give

5.19 $$\begin{aligned} \begin{pmatrix} x_j \\ y_j\end{pmatrix}= &   (1-DE(\lambda _j))^{-1}\gamma \nonumber \\ z_j= &   a + \langle E(\lambda _j)(1-DE(\lambda _j))^{-1}\gamma ,\beta \rangle . \end{aligned}$$

Now define $\varphi \in \textrm{Hol}(R_\delta )$ by

5.20 $$\begin{aligned} \varphi (\lambda ) = a + \langle E(\lambda )(1-DE(\lambda ))^{-1}\gamma ,\beta \rangle _{\mathbb {C}^{r_1} \oplus \mathbb {C}^{r_2}}, \ \text {for} \ \lambda \in R_\delta . \end{aligned}$$

By equation (5.19), $\varphi (\lambda _j) = z_j$ for $j=1,\dots ,n$, and by [6, Theorem 9.54], $\varphi \in \mathcal {S}_{\textrm{dp}}$, while equation (5.20) constitutes a DP-realization for $\varphi$. By Cramer’s rule for an invertible matrix, the function $\varphi$ defined by equation (5.20) is a rational function. Accordingly, by Theorem 3.8, if we set $\mathcal {M}=\mathbb {C}^{r_1} \oplus \mathbb {C}^{r_2}$ and define a holomorphic function $u: R_\delta \rightarrow \mathcal {M}$ by $u(\lambda )=(1-E(\lambda )D)^{-1}\gamma$, for $\lambda \in R_\delta$, then $(\mathcal {M},u)$ as in equation (5.14) is a DP-model for $\varphi$, while clearly $\dim \mathcal {M}=r_1+r_2 \le 2n$. $\square$

### Remark 5.21

*Solvable Pick data on *$\mathbb {D}$ *are also solvable as DP Pick data.* Let $\lambda _1,\ldots ,\lambda _n$ be *n* distinct points in $R_\delta$ and $z_1,\ldots ,z_n \in \mathbb {C}$. Suppose the Pick interpolation problem on the open unit disc $\mathbb {D}$

$$ \lambda _j \mapsto z_j \quad \text {for} \ j=1,\ldots ,n $$

is solvable. Then the DP Pick Problem

$$ \lambda _j \mapsto z_j \quad \text {for} \ j=1,\ldots ,n $$

is also solvable.

### Proof

By the assumption, there exists a holomorphic function $\varphi :\mathbb {D}\rightarrow \mathbb {C}$ such that $\varphi (\lambda _j)=z_j$ for $j=1,\dots ,n$ and $\Vert \varphi \Vert _{\textrm{H}^\infty (\mathbb {D})}\le 1$. By Proposition 2.11,

5.22 $$\begin{aligned} \Vert \varphi |R_\delta \Vert _{\textrm{dp}} =\Vert \varphi \Vert _{\textrm{H}^\infty (\mathbb {D})} \le 1, \end{aligned}$$

and so the restriction of $\varphi$ to $R_\delta$ is in $\mathcal {S}_{\textrm{dp}}$, which is to say that the corresponding DP Pick problem is solvable. $\square$

As the dp norm and sup norm are different, the converse statement to Remark 5.21 is false, as one would expect. The following two examples provide concrete instances of this fact.

### Example 5.23

*A solvable DP Pick data-set which is not a solvable Pick data-set on *$\mathbb {D}$.

Let $\delta \in (0,\tfrac{1}{2})$ and consider the 2 distinct points $\lambda _1 =\tfrac{1}{2}, \lambda _2 = -\tfrac{1}{2}$ in $R_\delta$. Recall that in Example 2.9 we showed that the function $\varphi \in \textrm{Hol}(R_\delta )$, $\varphi (\lambda ) = \tfrac{1}{2}(\lambda + \frac{\delta }{\lambda })$ satisfies $\Vert \varphi \Vert _{\textrm{dp}} =1$. Let $z_1=\varphi (\lambda _1)= \delta + \frac{1}{4}$, $z_2=\varphi (\lambda _2)= -(\delta + \frac{1}{4})$. Thus the DP Pick Problem

$$ \lambda _j \mapsto z_j \quad \text {for} \ j=1,2, $$

is solvable by the function $\varphi (\lambda ) = \tfrac{1}{2}(\lambda + \frac{\delta }{\lambda })$.

As to the Pick interpolation problem on the open unit disc $\mathbb {D}$

$$ \lambda _j \mapsto z_j \quad \text {for} \ j=1,2, $$

solvability depends on the value of $\delta$. There are 3 cases:

(i) for $\delta \in (0,\frac{1}{4})$, the Pick interpolation problem
  $$ \lambda _j \mapsto z_j \quad \text {for} \ j=1,2, $$
  is solvable;
(ii) for $\delta = \frac{1}{4}$, the Pick interpolation problem
  $$ \lambda _j \mapsto z_j \quad \text {for} \ j=1,2, $$
  on the open unit disc $\mathbb {D}$ is extremally solvable and has the unique solution $f(\lambda ) = \lambda$;
(iii) for $\delta \in (\frac{1}{4},\frac{1}{2} )$, the Pick interpolation problem
  $$ \lambda _j \mapsto z_j \quad \text {for} \ j=1,2, $$
  is not solvable on $\mathbb {D}$.

### Proof

To prove (i)-(iii) on the solvability of the Pick interpolation problem on the open unit disc $\mathbb {D}$

$$ \lambda _j \mapsto z_j \quad \text {for} \ j=1,2, $$

we consider the appropriate Pick matrix, which here is

$$ P(\delta ) = \displaystyle \begin{bmatrix} \frac{1-\overline{z_i} z_j}{1-\overline{\lambda _i}\lambda _j} \end{bmatrix}_{i,j=1}^2. $$

That is,

5.24 $$\begin{aligned} P(\delta ) =\begin{bmatrix} \frac{1-(\delta + \frac{1}{4})^2}{ 1- \frac{1}{4}} &  \frac{1+(\delta + \frac{1}{4})^2}{ 1+ \frac{1}{4}} \\ \\ \frac{1+(\delta + \frac{1}{4})^2}{ 1+ \frac{1}{4}} &  \frac{1-(\delta + \frac{1}{4})^2}{ 1- \frac{1}{4}}.\\ \end{bmatrix} \end{aligned}$$

It is clear that, for $\delta \in (0,\tfrac{1}{2})$,

$$ P(\delta )_{11} =\frac{1-(\delta + \frac{1}{4})^2}{ 1- \frac{1}{4}} > 0. $$

A little calculation shows that the determinant of the Pick matrix

$$ \det P(\delta )= \frac{16^2}{15^2}\left\{ \delta ^2+\tfrac{1}{2}\delta -\frac{3}{16}\right\} \left\{ \delta ^2+\tfrac{1}{2}\delta -\frac{63}{16}\right\} , $$

from which one can deduce that

(i) when $\delta \in (0,\frac{1}{4})$, $\det P(\delta ) >0$ and so $P(\delta )> 0$;
(ii) $\det P(\delta ) =0$, when $\delta = \frac{1}{4}$; and
(iii) $$\det P(\delta ) <0$$, when $$\delta \in (\frac{1}{4},\frac{1}{2} )$$.

Therefore the Pick matrix $P(\delta )$ is not positive when $\delta \in (\frac{1}{4},\frac{1}{2} )$. Thus, by Pick’s theorem, for $\delta \in (\frac{1}{4},\frac{1}{2} )$, the Pick interpolation problem

$$ \lambda _j \mapsto z_j \quad \text {for} \ j=1,2, $$

is not solvable, while, for $\delta =\tfrac{1}{4}$, the Pick interpolation problem is uniquely solvable, and one sees by inspection that the unique solution is the function $f(\lambda )=\lambda$. $\square$

### Example 5.25

*Another solvable DP Pick data-set which is not a solvable Pick data-set on *$\mathbb {D}$.

Let $\delta \in (0,1)$ and let $\lambda _1 = \frac{\delta +\sqrt{\delta }}{2}$, $\lambda _2=-\lambda _1$. We have $0<\delta< \frac{\delta +\sqrt{\delta }}{2}<\sqrt{\delta } <1$, so that $\lambda _1,\lambda _2 \in R_\delta$. Recall from Example 2.9 that the function $\varphi (z)=\tfrac{1}{2}(z+\frac{\delta }{z})$ on $R_\delta$ satisfies $\Vert \varphi \Vert _{\textrm{dp}} =1$. Consider the DP-Pick problem

5.26 $$\begin{aligned} \lambda _i \mapsto z_i {\mathop {=}\limits ^\textrm{def}}\varphi (\lambda _i), i=1,2. \end{aligned}$$

Clearly this is a solvable DP-Pick problem, with solution $\varphi$. However, the Pick problem with the same data $\lambda _j \mapsto z_j, j=1,2$, is not solvable. Indeed, the Pick matrix for the problem (5.26) is

$$ P= \begin{bmatrix} \frac{1-|z_1|^2}{1-|\lambda _1|^2} &  \frac{1+|z_1|^2}{1+|\lambda _1|^2} \\ \frac{1+|z_1|^2}{1+|\lambda _1|^2} &  \frac{1-|z_1|^2}{1-|\lambda _1|^2} \end{bmatrix}. $$

Thus

$$\begin{aligned} \det P&= \left( \frac{1-|z_1|^2}{1-|\lambda _1|^2}\right) ^2 - \left( \frac{1+|z_1|^2}{1+|\lambda _1|^2}\right) ^2 \\&= D_1D_2 \end{aligned}$$

where

$$\begin{aligned} D_1&= \frac{1-|z_1|^2}{1-|\lambda _1|^2} - \frac{1+|z_1|^2}{1+|\lambda _1|^2} \\&= \frac{2(|\lambda _1|^2 - |z_1|^2)}{1-|\lambda _1|^4}, \\ D_2&= \frac{1-|z_1|^2}{1-|\lambda _1|^2} + \frac{1+|z_1|^2}{1+|\lambda _1|^2} \\&=\frac{2(1-|z_1\lambda _1|^2)}{1-|\lambda _1|^4}. \end{aligned}$$

Now

$$\begin{aligned} z_1&= \frac{1}{2} \left( \lambda _1+\frac{\delta }{\lambda _1} \right) = \frac{1}{2}\left( \frac{\delta +\sqrt{\delta }}{2} + \frac{2\delta }{\delta +\sqrt{\delta }}\right) \\&= \frac{\sqrt{\delta }}{2}\left( \frac{1+\sqrt{\delta }}{2} + \frac{2}{1+\sqrt{\delta }}\right) = \frac{\sqrt{\delta }(5+2\sqrt{\delta } +\delta )}{4(1+\sqrt{\delta })}, \end{aligned}$$

and

$$\begin{aligned} 0< z_1\lambda _1&= \frac{\sqrt{\delta }(5+2\sqrt{\delta } +\delta )}{4(1+\sqrt{\delta })}. \frac{\sqrt{\delta }(1+\sqrt{\delta })}{2} = \frac{\delta (5+2\sqrt{\delta } +\delta )}{8} \\&< 1. \end{aligned}$$

Thus $D_2 > 0$, and moreover

$$\begin{aligned} |\lambda _1| - |z_1|&= \lambda _1- \tfrac{1}{2}(\lambda _1 + \frac{\delta }{\lambda _1}) = \tfrac{1}{2}\left( \frac{\delta +\sqrt{\delta }}{2} - \frac{2\delta }{\delta +\sqrt{\delta }} \right) \\&= \frac{\sqrt{\delta }}{2}\left( \frac{1+\sqrt{\delta }}{2} - \frac{2}{1+\sqrt{\delta }}\right) \\&= \frac{\sqrt{\delta }(-3+2\sqrt{\delta }+\delta )}{4(1+\sqrt{\delta })} \\&< 0, \end{aligned}$$

from which it follows that $D_1 < 0$, and hence $D < 0$. Thus the Pick matrix *P* is not positive, and so, by Pick’s Theorem, the Pick interpolation problem $\lambda _j \mapsto z_j, j=1,2$, is not solvable.

Since the Pick interpolation problem on $\mathbb {D}$ and the DP Pick problem on $R_\delta$ are so closely related, it is natural to ask whether the Szegő kernel on $\mathbb {D}$, when retricted to $R_\delta$, is a DP-Szegő kernel. We can use Example 5.25 to answer this question in the negative.

### Proposition 5.27

Let $\delta \in (0,1)$. The Szegő kernel $[\frac{1}{1-{\overline{\mu }} \lambda }]$ restricted to $R_\delta$ is not a DP Szegő kernel on $R_\delta$.

### Proof

Suppose the kernel $[\frac{1}{1-{\overline{\mu }} \lambda }]$ restricted to $R_\delta$ is a DP kernel. Then, for any distinct $\lambda _1,\dots ,\lambda _n \in R_\delta$, the localization of $[\frac{1}{1-{\overline{\mu }} \lambda }]$ to $\{\lambda _1,\dots ,\lambda _n \}$ belongs to $\mathcal {G}_{\textrm{dp}} (\lambda )$.

Consider the 2 distinct points $$\lambda _1 = \frac{\delta +\sqrt{\delta }}{2}$$, $$\lambda _2=-\lambda _1$$, note that, for $$\delta \in (0,1)$$, $$\lambda _1,\lambda _2 \in R_\delta $$. By Example 2.9, the function $$\varphi (z)=\tfrac{1}{2}(z+\frac{\delta }{z})$$ on $$R_\delta $$ satisfies $$\Vert \varphi \Vert _{\textrm{dp}} =1$$. Therefore, for $$\lambda _i$$ and $$z_i= \varphi (\lambda _i)= \frac{1}{2} \left( \lambda _i+\frac{\delta }{\lambda _i} \right) $$, $$ i=1,2$$, the DP Pick problem

5.28 $$\begin{aligned} \lambda _j \mapsto z_j \quad \text {for} \ j=1,2, \end{aligned}$$

is solvable. In Example 5.25 we showed that, for $\delta \in (0,1)$, the corresponding Pick problem (5.28) is not solvable.

Since the problem (5.28) is a solvable DP Pick problem, by Theorem 5.2, for all $g \in \mathcal {G}_{\textrm{dp}} (\lambda )$,

5.29 $$\begin{aligned} {[}(1-{\overline{z}}_i z_j)g_{ij}] \ge 0. \end{aligned}$$

By the assumption, the localization of $[\frac{1}{1-{\overline{\mu }} \lambda }]$ to $\{\lambda _1,\lambda _2 \}$ belongs to $\mathcal {G}_{\textrm{dp}} (\lambda )$. In particular, for the Pick problem

5.30 $$\begin{aligned} \lambda _j \mapsto z_j \quad \text {for} \ j=1,2, \end{aligned}$$

on $\mathbb {D}$, the Pick matrix

5.31 $$\begin{aligned} \left[ \frac{1-{\overline{z}}_i z_j}{1-{\overline{\lambda }}_i\lambda _j}\right] _{i,j=1}^2 \ge 0. \end{aligned}$$

Hence, by Pick’s theorem, the problem (5.30) is solvable by a Schur function *f* on $\mathbb {D}$. This contradicts our example, and so $[\frac{1}{1-{\overline{\mu }} \lambda }]$ is not a DP Szegő kernel on $R_\delta$. $\square$

## Extremal DP Pick problems

In this section we study DP Pick interpolation problems that are “only just” solvable. We say that a DP Pick problem is *extremally solvable* if it is solvable and there does not exist $\varphi \in \textrm{H}^\infty _{\textrm{dp}}$ with $\Vert \varphi \Vert _{\textrm{dp}} <1$ satisfying the equations

6.1 $$\begin{aligned} \varphi (\lambda _j) = z_j \qquad \text {for} \ j=1,\ldots ,n. \end{aligned}$$

### Remark 6.2

A DP Pick problem that is not extremally solvable cannot have a unique solution. For suppose $\lambda _j \mapsto z_j, j=1,\dots ,n,$ is a solvable DP Pick problem that is not extremally solvable. That means that there is a function $\varphi :R_\delta \rightarrow \mathbb {C}$ such that $\varphi (\lambda _j)=z_j$ for $j=1, \dots ,n$ and $\Vert \varphi \Vert _{\textrm{dp}}<1$. Consider the function $\psi (\lambda )=\varphi (\lambda ) + \varepsilon \prod _{j=1}^n (\lambda -\lambda _j)$, for $\lambda \in R_\delta$, for some positive $\varepsilon$. Then $\psi (\lambda _j)=z_j$ for $j=1, \dots ,n$ and

$$\Vert \psi \Vert _{\textrm{dp}}\le \Vert \varphi \Vert _{\textrm{dp}} + \varepsilon \Vert \prod _{j=1}^n (\lambda -\lambda _j)\Vert _{\textrm{dp}}<1$$

for all small enough $\varepsilon$, and so there are infinitely many solutions to the interpolation problem $\lambda _j \mapsto z_j$ for $j=1,\dots ,n$ having DP norm less than 1.

Next we give necessary and sufficient conditions for a DP Pick problem to be extremally solvable.

### Theorem 6.3

Let $\lambda _1,\dots ,\lambda _n \in R_\delta$ be distinct and let $z_1,\dots ,z_n\in \mathbb {C}$. The following two statements are equivalent.

(i) The DP Pick problem
  $$ \lambda _j \mapsto z_j \quad \text {for} \ j=1,\ldots ,n $$
  is extremally solvable.
(ii) For all $g \in \mathcal {G}_{\textrm{dp}} (\lambda )$,
  6.4 $$\begin{aligned} {[}(1-{\overline{z}}_i z_j)g_{ij}]_{i,j=1}^n \ge 0 \end{aligned}$$
  and there exists $\widetilde{g}\in \mathcal {G}_{\textrm{dp}}(\lambda )$ such that
  6.5 $$\begin{aligned} {{\,\textrm{rank}\,}}\big [(1-{\overline{z}}_i z_j)\widetilde{g}_{ij}\big ]_{i,j=1}^n < n. \end{aligned}$$

### Proof

(i) $\implies$ (ii). Suppose that the DP Pick problem

$$ \lambda _j \mapsto z_j\ \text {for}\ j=1,\dots ,n $$

is extremally solvable. Since the problem is solvable, Theorem 5.2 implies that, for all $g \in \mathcal {G}_{\textrm{dp}} (\lambda )$,

6.6 $$\begin{aligned} {[}(1-{\overline{z}}_i z_j)g_{ij}] \ge 0. \end{aligned}$$

Suppose, for a contradiction, that there is no $g \in \mathcal {G}_{\textrm{dp}} (\lambda )$ such that

6.7 $$\begin{aligned} {[}(1-{\overline{z}}_i z_j)g_{ij}]\ \text {is singular}. \end{aligned}$$

Let $F:\mathbb {R}\times \mathcal {G}_{\textrm{dp}}^{\textrm{norm}} (\lambda ) \rightarrow \mathbb {R}$ be defined by

$$\begin{aligned} F(r,[g_{ij}])&= \text {the minimum of the leading principal minors of} \\  &\quad \begin{bmatrix} (1-r^2{\overline{z}}_i z_j) g_{ij}\end{bmatrix}_{i,j=1}^n \nonumber \\&=\min _{J=1,\dots ,n} \det \begin{bmatrix} (1-r^2{\overline{z}}_i z_j) g_{ij}\end{bmatrix}_{i,j=1}^J. \end{aligned}$$

By standard linear algebra, for any positive definite matrix $g=[g_{ij}]$, $F(r,g)> 0$ if and only if $$\begin{bmatrix} (1-r^2{\overline{z}}_i z_j) g_{ij}\end{bmatrix}_{i,j=1}^n > 0$$. *F* is continuous and, by supposition,

$$ {[}(1-{\overline{z}}_i z_j)g_{ij}] > 0$$

for all $g \in \mathcal {G}_{\textrm{dp}} (\lambda )$, which implies that $F(1,g) > 0$ for all $g \in \mathcal {G}_{\textrm{dp}}^{\textrm{norm}} (\lambda )$. Since, by Proposition 4.23, $\mathcal {G}_{\textrm{dp}}^{\textrm{norm}} (\lambda )$ is compact, $F(1,\cdot )$ attains its minimum on $\mathcal {G}_{\textrm{dp}}^{\textrm{norm}} (\lambda )$, and so there exists $\kappa >0$ such that $F(1,g)\ge \kappa$ for all $g\in \mathcal {G}_{\textrm{dp}}^{\textrm{norm}} (\lambda )$.

By the continuity of *F* and, again by the compactness of $\mathcal {G}_{\textrm{dp}}^{\textrm{norm}}(\lambda )$, the family of functions $\{ F(\cdot ,g):g \in \mathcal {G}_{\textrm{dp}}^{\textrm{norm}} (\lambda )\}$ is equicontinuous on $\mathbb {R}$. Hence there exists $\delta > 0$ such that $F(r,g) > 0$ for all $g\in \mathcal {G}_{\textrm{dp}}^{\textrm{norm}} (\lambda )$ and all $r\in (1,\delta )$. Choose some $r\in (1,\delta )$. Then $F(r,g) > 0$ for all $g\in \mathcal {G}_{\textrm{dp}}^{\textrm{norm}} (\lambda )$, and therefore $$\begin{bmatrix} (1-r^2{\overline{z}}_i z_j) g_{ij}\end{bmatrix}_{i,j=1}^n > 0$$ for all $g\in \mathcal {G}_{\textrm{dp}}^{\textrm{norm}} (\lambda )$. It follows from Proposition 4.23 that $$\begin{bmatrix} (1-r^2{\overline{z}}_i z_j) g_{ij}\end{bmatrix}_{i,j=1}^n > 0$$ for all $g\in \mathcal {G}_{\textrm{dp}} (\lambda )$. Hence, by Theorem 5.2, for the chosen $r\in (1,\delta )$, the DP Pick problem

$$ \lambda _j \mapsto rz_j\ \text {for}\ j=1,\dots ,n $$

is solvable, which is to say that there exists a function $\psi \in \textrm{Hol}(R_\delta )$ such that $\Vert \psi \Vert _{\textrm{dp}} \le 1$ and $\psi (\lambda _j)=rz_j$ for $j=1,\dots ,n$. Thus the function $\varphi {\mathop {=}\limits ^\textrm{def}}\psi /r$ satisfies $\varphi (\lambda _j)=z_j$ for $j=1,\dots ,n$ and $\Vert \varphi \Vert _{\textrm{dp}} \le 1/r < 1$, contrary to hypothesis. Hence there is a $g \in \mathcal {G}_{\textrm{dp}} (\lambda )$ such that

6.8 $$\begin{aligned} {[}(1-{\overline{z}}_i z_j)g_{ij}]\ \text {is singular}. \end{aligned}$$

We have shown that statements (6.4) and (6.5) hold, and so have established (i) $\Rightarrow$ (ii) in Theorem 6.3.

(ii) $\Rightarrow$ (i). Suppose that (ii) holds, and so, for all $g \in \mathcal {G}_{\textrm{dp}} (\lambda )$,

6.9 $$\begin{aligned} {[}(1-{\overline{z}}_i z_j)g_{ij}]_{i,j=1}^n \ge 0. \end{aligned}$$

By Theorem 5.2, there exists $\varphi \in \mathcal {S}_{\textrm{dp}}$ such that

6.10 $$\begin{aligned} \varphi (\lambda _j) = z_j \qquad \text {for} \ j=1,\ldots ,n. \end{aligned}$$

Suppose (i) does not hold, which means that the problem is non-extremally solvable, and hence there exists $\varphi$ such that $\Vert \varphi \Vert _{\textrm{dp}} = r < 1$ and $\varphi$ satisfies $\varphi (\lambda _j)=z_j$ for $j=1,\dots ,n$. Thus for all $g \in \mathcal {G}_{\textrm{dp}} (\lambda )$,

6.11 $$\begin{aligned} {[}(r^2-\overline{z_i}z_j)g_{ij}]\ge 0. \end{aligned}$$

By assumption (ii), there exists $\widetilde{g}\in \mathcal {G}_{\textrm{dp}}(\lambda )$ such that

6.12 $$\begin{aligned} {{\,\textrm{rank}\,}}\big [(1-{\overline{z}}_i z_j)\widetilde{g}_{ij}\big ]_{i,j=1}^n < n. \end{aligned}$$

and hence $[(1-{\overline{z}}_i z_j)\widetilde{g}_{ij}]_{i,j=1}^n$ has a non-zero null vector *v*. Consider the relation

$$ (1-{\overline{z}}_i z_j) {\widetilde{g}}_{ij} = (1-r^2) {\widetilde{g}}_{ij} +(r^2-{\overline{z}}_i z_j) {\widetilde{g}}_{ij}. $$

Since ${\widetilde{g}}_{ij}>0$, $(1-r^2) {\widetilde{g}}_{ij}>0$ and, by equation (6.11), $(r^2-{\overline{z}}_i z_j) {\widetilde{g}}_{ij} \ge 0$, which is a contradiction. $\square$

By Theorem 5.12, if a DP Pick problem is solvable, then there exists a rational solution $\varphi \in \mathcal {S}_{\textrm{dp}}$. In the next theorem we show that if, further, the problem is *extremally* solvable then there exists $T\in \mathcal {F}_{\textrm{dp}}(\delta ,\lambda )$, acting on an *n*-dimensional Hilbert space, such that $\Vert \varphi (T) \Vert =\Vert \varphi \Vert _{\textrm{dp}} = 1.$

### Theorem 6.13

Let $\lambda _1,\dots ,\lambda _n \in R_\delta$ be distinct and let $z_1,\dots ,z_n\in \mathbb {C}$. If the DP Pick problem

$$ \lambda _j \mapsto z_j \quad \text {for} \ j=1,\ldots ,n, $$

is extremally solvable, then there exists a rational function $\varphi \in \mathcal {S}_{\textrm{dp}}$ which satisfies the equations

6.14 $$\begin{aligned} \varphi (\lambda _j) = z_j \qquad \text {for} \ j=1,\ldots ,n, \end{aligned}$$

and has a model $(\mathcal {M},u)$ as in equation (5.14), where $u: R_\delta \rightarrow \mathcal {M}$ is a holomorphic function and $\dim \mathcal {M}\le 2n$. Furthermore, there exists $T\in \mathcal {F}_{\textrm{dp}}(\delta ,\lambda )$ such that

$$ 1=\Vert \varphi \Vert _{\textrm{dp}} = \Vert \varphi (T) \Vert . $$

In particular,

$$ 1-\varphi (T)^*\varphi (T) = u(T)^*\big (1-E(T)^*E(T)\big )\ u(T), $$

where $\mathcal {M}$ can be written as $\mathcal {M}=\mathcal {M}_1\oplus \mathcal {M}_2$ and $E:R_\delta \rightarrow \mathcal {B}(\mathcal {M})$ is defined by the formula

6.15 $$\begin{aligned} E(\lambda )=\begin{bmatrix}\lambda &  0\\ 0 &  \frac{\delta }{\lambda }\end{bmatrix}\ \qquad \text {for}\ \lambda \in R_\delta \end{aligned}$$

with respect to this orthogonal decomposition of $\mathcal {M}$.

### Proof

Since the DP Pick problem $\lambda _j \mapsto z_j, \ \text {for} \ j=1,\ldots ,n,$ is solvable, by Theorem 5.12, there exists a rational function $\varphi \in \mathcal {S}_{\textrm{dp}}$ such that $\varphi (\lambda _j) = z_j, \text {for} \ j=1,\ldots ,n$.

Let us now prove the existence of an operator *T* with the stated properties. By assumption, the DP-Pick problem $\lambda _j \mapsto z_j, j=1,\dots ,n$ is *extremally* solvable. By Theorem 6.3, there exists ${\widetilde{g}} \in \mathcal {G}_{\textrm{dp}}(\lambda )$ such that

6.16 $$\begin{aligned} {{\,\textrm{rank}\,}}\big [(1-{\overline{z}}_i z_j)\widetilde{g}_{ij}\big ]_{i,j=1}^n < n, \end{aligned}$$

so that $$\begin{bmatrix} (1-{\overline{z}}_i z_j)\widetilde{g}_{ij} \end{bmatrix}$$ is singular, and therefore has a non-zero null vector $\xi =[\xi _1,\dots ,\xi _n]^T \in \mathbb {C}^n$, which is to say that

6.17 $$\begin{aligned} \sum _{j=1}^n (1-{\overline{z}}_i z_j)\widetilde{g}_{ij} \xi _j = 0\ \text {for}\ i=1,\dots ,n. \end{aligned}$$

Since ${\widetilde{g}} \in \mathcal {G}_{\textrm{dp}}(\lambda )$, $[{\widetilde{g}}_{ij}] > 0$, and so $[{\widetilde{g}}_{ij}]$ has rank *n*. By Moore’s Theorem there exist an *n*-dimensional Hilbert space $\mathcal {H}$ and a basis ${\widetilde{e}}_1, \dots ,{\widetilde{e}}_n \in \mathcal {H}$ such that ${\widetilde{g}}_{ij}=\langle {\widetilde{e}}_j,{\widetilde{e}}_i\rangle$ for $i,j=1,\dots ,n$.

Define an operator *T* on $\mathcal {H}$ by $T{\widetilde{e}}_j = \lambda _j{\widetilde{e}}_j$ for $j=1,\dots ,n$. Since ${\widetilde{g}} \in \mathcal {G}_{\textrm{dp}}(\lambda )$, by Proposition 4.10, $T\in \mathcal {F}_{\textrm{dp}}(\delta ,\lambda )$. Note that $\varphi (T)\widetilde{e}_j=z_j{\widetilde{e}}_j$, and so, if $x=\sum _{j=1}^n \xi _j\widetilde{e}_j$, then

$$ \varphi (T)x = \sum _{j=1}^n z_j\xi _j{\widetilde{e}}_j $$

and

6.18 $$\begin{aligned} \langle (1-\varphi (T)^*\varphi (T))x,x\rangle= &   \sum _{i,j=1}^n (1-{\overline{z}}_iz_j) {\overline{\xi }}_i \xi _j\langle {\widetilde{e}}_j,{\widetilde{e}}_i\rangle \nonumber \\  &   =\sum _{i,j=1}^n (1-{\overline{z}}_iz_j) {\overline{\xi }}_i \xi _j\widetilde{g}_{ij} \nonumber \\  &   = \sum _{i=1}^n {\overline{\xi }}_i\sum _{j=1}^n (1-{\overline{z}}_iz_j) \xi _j{\widetilde{g}}_{ij} \nonumber \\  &   = 0. \end{aligned}$$

As $\xi \ne 0$, the complex numbers $\xi _1,\dots ,\xi _n$ are not all zero, and so, since ${\widetilde{e}}_1,\dots ,{\widetilde{e}}_n$ are linearly independent, $x=\sum _{j=1}^n \xi _j{\widetilde{e}}_j \ne 0$. Since $\Vert \varphi \Vert _{\textrm{dp}} \le 1$ and $T\in \mathcal {F}_{\textrm{dp}}(\delta ,\lambda )$, we have $\Vert \varphi (T)\Vert \le 1$, and so $1-\varphi (T)^*\varphi (T) \ge 0$. In conjunction with the equality (6.18), this implies that $(1-\varphi (T)^*\varphi (T))x=0$, and hence $\Vert \varphi (T)x\Vert ^2=\Vert x\Vert ^2$. Since $x\ne 0$, *x* is a maximizing vector for $\varphi (T)$ and $\Vert \varphi (T)\Vert =1$.

By Theorem 5.12, for the rational function $\varphi$ there exists a model $(\mathcal {M}, u),$ where $u: R_\delta \rightarrow \mathcal {M}$ is holomorphic, so that

6.19 $$\begin{aligned} 1-\overline{\varphi (\mu )}\varphi (\lambda ) = \Big \langle \, \big (1-E(\mu )^*E(\lambda )\big )\ u(\lambda )\, ,u(\mu )\, \Big \rangle _\mathcal {M}\ \text {for}\ \lambda ,\mu \in R_\delta , \end{aligned}$$

where $\dim \mathcal {M}\le 2n$. Since $T\in \mathcal {F}_{\textrm{dp}}(\delta ,\lambda )$, *T* satisfies

$$ \sigma (T)=\{\lambda _1,\ldots ,\lambda _n\} \subset R_\delta . $$

Thus, by the Riesz-Dunford functional calculus, $\varphi (T)$ is well defined and, by the hereditary functional calculus,

$$ 1-\varphi (T)^*\varphi (T) = u(T)^*\big (1-E(T)^*E(T)\big )\ u(T). $$

$$\square $$

## Data Availability

No data were collected, generated or consulted in connection with this research.

## Footnotes

1 $\Vert \cdot \Vert _{\textrm{dp}}$ is an example of a *calcular norm*, see [6, Chapter 9]

2 In the notations $\Vert \cdot \Vert _{\textrm{dp}}$ and $\mathcal {S}_{\textrm{dp}}$ we suppress dependence on the parameter $\delta$.

3 A *calcular norm* on a function space is a norm that is defined with the aid of the functional calculus. For more information on such norms the reader may consult [6, Chapter 9].

4 In the notations $\Vert \cdot \Vert _{\textrm{bfd}}$ and $\mathcal {S}_{\textrm{bfd}}$ we suppress dependence on the parameter $\delta$.

5 Equivalently, $$\{ u(\lambda ) \, : \, \lambda \in \Omega \}$$ spans $$\mathcal {M}$$.
